# Effects of Age and Cognition on a Cross-Cultural Paediatric Adaptation of the Sniffin' Sticks Identification Test

**DOI:** 10.1371/journal.pone.0131641

**Published:** 2015-08-12

**Authors:** Laís Orrico Donnabella Bastos, Marilisa Mantovani Guerreiro, Andrew John Lees, Thomas T. Warner, Laura Silveira-Moriyama

**Affiliations:** 1 Complex motor disorders service, Neurology Department and Physical and Occupational Therapy Department, HC-FCM, University of Campinas, UNICAMP, Campinas, São Paulo, Brazil; 2 Neurology Department, HC-FCM, University of Campinas, UNICAMP, Campinas, São Paulo, Brazil; 3 Reta Lila Weston Institute of Neurological Studies, UCL Institute of Neurology, London, United Kingdom; 4 Medicine Department, Universidade Nove de Julho, Uninove, São Paulo, São Paulo, Brazil; Université Lyon, FRANCE

## Abstract

**Objectives:**

To study the effects of age and cognition on the performance of children aged 3 to 18 years on a culturally adapted version of the 16 item smell identification test from Sniffin' Sticks (SS16).

**Methods:**

A series of pilots were conducted on 29 children aged 3 to 18 years old and 23 adults to produce an adapted version of the SS16 suitable for Brazilian children (SS16-Child). A final version was applied to 51 children alongside a picture identification test (PIT-SS16-Child) to access cognitive abilities involved in the smell identification task. In addition 20 adults performed the same tasks as a comparison group.

**Results:**

The final adapted SS16-Child was applied to 51 children with a mean age of 9.9 years (range 3-18 years, SD=4.25 years), of which 68.3% were girls. There was an independent effect of age (p<0.05) and PIT-SS16-Child (p<0.001) on the performance on the SS16-Child, and older children reached the ceiling for scoring in the cognitive and olfactory test. Pre-school children had difficulties identifying items of the test.

**Discussion/Conclusions:**

A cross-culturally adapted version of the SS16 can be used to test olfaction in children but interpretation of the results must take age and cognitive abilities into consideration.

## Introduction

Various clinical diseases which manifest in the adult life are known to present with olfactory loss, including Parkinson’s disease and Alzheimer’s type dementia, which both present with significant smell loss in more than 70% of patients [1]. Nevertheless, until fairly recently there was scarce literature on smell testing in children, and currently little is known about the occurrence of smell loss in neuropaediatric conditions. The limited literature on smell testing in children suggests that adult tests might be adapted to children, and that there is an association between children’s age and performance in the test with older children scoring higher [2–18], but there is no consensus on the minimum age limit for the application of smell identification tests.

Three main modalities of smell tests are used in the clinical and research practice: odour identification, odour discrimination and odour detection threshold [19–22]. The most common task of odour detection threshold is the 3-alternative forced-choice in which the subject must detect which of three pens (or tubes) contains an odorant. The odorant comes in different concentrations and the threshold is the minimal concentration at which reliable detection is observed. In the tests of odour discrimination, the subject is again presented with triplets, and needs to discriminate which one contains a smell that is different from the other two. Smell identification is usually a forced choice task in which the subject must identify an odorant among a given number of written options (generally four). Although this is the most practical modality in the clinical setting due to ease of application, it is nevertheless more affected by familiarity with the items. This renders identification tasks more sensitive to cultural differences and careful adaptation and cultural validation is needed before it can be applied cross-culturally. In theory, this modality is strongly influenced by the olfactory learning which happens in children and significant adaptations are needed before a test which was developed for adults can be applied to a paediatric population.

The two most commonly used smell identification tests worldwide are the University of Pennsylvania Smell Identification Test (UPSIT) [23] and the smell identification test from Sniffin' Sticks [24], both of which have been cross-culturally adapted for use in different countries, but only in the adult population. No smell test has been adapted for the Brazilian paediatric population to date. The University of Pennsylvania Smell Identification test has had two different adaptations to the Brazilian population yielding a final one which can be reliably applied to Brazilian adults [25,26], but no world-wide adaptation of this test exists for children. The 16 item smell identification test from Sniffin' Sticks (SS16) has been successfully adapted for the use in Brazilian adults [27].

The development of olfactory tests for children occurred at a slower rate than for the adult population. In contrast, the normative data for more than 2000 adults have been available for the University of Pennsylvania Smell Identification Test in the US [23,28] for over 20 years, but the normative data for a pediatric test from the same group was only made available in 2013 [13]. Large European studies provided normative data for adult Europeans using the smell test battery form the Sniffin' Sticks (SS16) in 2007[29], and only recently Schriever et al [15] produced an adaptation of the 16 item smell identification test from Sniffin Sticks to German children older than 6 years, showing that 14 of the 16 items were well recognized by children (a subscale they called "Sniffin Kids"). A small number of studies evaluated the applicability of smell identification tests to children, and ours is the first to include subjects outside of Europe, North-America or Australia. Cultural factors strongly influence the performance on odour identification tests [26] and therefore in order to apply the SS16 or the “Sniffin Kids” to Brazilian children, modifications are likely to be necessary.

In addition to the influence of cultural factors on the performance of smell tests, cognitive status is a known determinant of smell identification performance in adults [22,30,31]. Nevertheless, the relationship between cognition and olfactory testing has not been systematically explored in children.

Thus, the objective of this study was to create an adapted version of SS16 suitable to a Brazilian paediatric population. A secondary objective was to examine how age and cognition (as measured by a simple cognitive screening task) affected the performance of children and therefore the interpretation of test results. This new version (called by us SS16-Child) was applied in children aged 3 to 18 years old alongside a cognitive test adapted from the Picture Identification Test (PIT), which is used for cognitive screening in adult populations who undergo the UPSIT [32].

## Materials and Methods

### Ethics Statement

The study was approved by the Research and Ethics Committee of State University of Campinas (Comitê de Ética em Pesquisa da Faculdade de Ciências Médicas da Unicamp, CEP-FCM-UNICAMP) and was registered in the national registry at the National Commission for Ethics in Research (Comissão Nacional de Ética em Pesquisa, CONEP) under the number CAAE: 06852212.4.0000.5404. Written informed consent was obtained from adult participants. For participants aged 18 or younger verbal informed assent was obtained from the participant and written informed consent was obtained from a parent or legal guardian on behalf of the participant, as approved by the CEP-FCM-UNICAMP.

### Subjects

A convenience sample was recruited amongst healthy adults and children who visited Hospital de Clínicas (UNICAMP, Brazil) accompanying adult patients or members of staff. The inclusion criteria were (1) willingness to participate and (a) informed consent from the adult participants, or (b) informed consent from parents or legal guardians combined with assent from participant, (2) clinical judgement of being neurologically normal based on an interview with the subjects or with the parents (in the case of small children), (3) ability to recognize at least 70% (11/16) of items of the picture identification test, therefore clearly demonstrating ability to understand the identification task and furthermore showing familiarity with at least 70% of items in the test. For the final study none of the 51 children fulfilling criteria (2) failed on criteria (3).

Exclusion criteria were a) known or suspected neurological or psychiatric disease; b) active upper respiratory tract infection or acute rhinitis in the day of evaluation. In order to check for exclusion criteria a brief questionnaire modified from Fornazieri et al [33] was applied. The initial pilots were tested in 29 children (mean age±SD: 9.4±4.3 years) and 23 adults (40±15.5 years) for comparison, and the final test was applied to 51 children (9.9±4.2 years) and 20 adults (39.4±10.7 years).

### Data Collection

The participant's assessment was performed in three sequential stages: (1) application of socioeconomic and health questionnaire (described in "demographic data"), (2) smell test and (3) cognitive test. The order was thus established because the questionnaire was needed to screen for inclusion and exclusion criteria, and then the cognitive test was performed last to avoid influencing recognition of the items in the smell test.

### Demographic Data

For all subjects we collected age, gender, socioeconomic level, health conditions and schooling. The socioeconomic level was assessed as previously described [25,26] by a question about the family income (average family income in comparison with the Brazilian minimum wage). Minimum wage is defined as the lowest monthly remuneration legally stipulated that the employer must pay to the employee. This value is updated regularly by the Brazilian government to reflect changes in cost-of-living [34,35]. Based on the answer to this question, subjects were further classified in the groups A (monthly family income of less than 5 times the value of the standardized Brazilian minimum wage) or B (monthly family income higher than 5 times the value of the standardized Brazilian minimum wage).

### Cognitive Evaluation

Due to the variable cognitive profile of children aged 3 through 18 years, we opted for a cognitive screening using a modification of the UPSIT Picture Identification Test, adapted to the Sniffin' Sticks smell test (PIT-SS16-Child) in its Brazilian version. The PIT is used as a means of cognitive screening in adult populations who undergo the UPSIT [32]. The PIT-SS16-Child is composed of sixteen pictures, which are identical to the pictures used to identify the correct answers in each of the 16 items of the smell test. For each figure in the test, four options are read by the examiner, and the subject must recognize the word which describes the figure.

The 23 adults who participated in the pilot studies were screened using the validated Portuguese version of Mini Mental State Examination (MMSE) and all scored above the recommended cut-off of 24 points [36]. In addition, the 20 adult subjects who underwent pilot 4 also performed the PIT-SS16-Child.

### Smell Testing

Smell identification was tested using the standardized application of a commercially available and extensively validated smell identification test called "16-item Smell Identification Test from Sniffin Sticks", hereafter called SS16 in this manuscript. This test is composed of 16 standardized pens produced in Germany (Burghart Messtechnik company) which must be presented sequentially to the subject, who then identifies the smell by a forced choice amongst 4 alternative (one correct label and 3 distractors). Each pen is held approximately 2 centimetres from the subject's nose for no more than 3 or 4 seconds. The interval between the presentations of each odour is of roughly 30 seconds [24, 37]. The original version of SS16 was validated to Brazilian adults by our group in 2008 [27]. This Brazilian version was then used as the base for a child-friendly adaptation produced as described in the following sections:

1. Replacement of the words for pictures as alternatives for the forced choice. When necessary, the picture for the item was replaced by a picture of an equivalent item more familiar to children. Example: for the item “mint” a photo of mint flavoured candies was used;
2. Replacement of labels of smells unknown to children for an equivalent or similar item. Example: the odour of anise seed (in the original labelled “anise”) was labelled “toothpaste” due to its perceived similarity with the essences often used for toothpaste in Brazil, as reported by children during informal focus groups preceding our pilot studies;
3. Replacement of the unfamiliar distractors for other more familiar options;
4. Replacement of distractors which were felt to be too similar to the smell (ie: to the correct choice) of the item by distractors which were more contrasting. E.g.: for the item 16, “fish”, the distractors used were “strawberry, orange, rose”, which are quite contrasting to “fish”. The selection of contrasting distractors was done based on a subjective impression of the researchers based on informal focus groups with children before and during the pilot studies, and on the percentage of subjects who choose each one of the distractors. If a larger proportion of subjects chose one specific wrong distractor, that was considered indirect evidence of subjective similarity of that distractor and the item.
5. After each pilot study involving a smaller group of children, new changes were made for items in which difficulties were demonstrated objectively (less than 75% identifiability) or reported subjectively (children referred that the item was confusing, etc).

## Results

The characteristics of the children enrolled in the four pilots of the study are shown in the first three columns of Table 1. The progressive changes made on the adaptation process can be seen in detail S1 Table) which shows how the labels for each of the items and distractors changed in the adaptation process.

**Table 1:** Demographics of children.

	Pilot 1	Pilot 2	Pilot 3	Final Study	TOTAL
**Number of participants**	N = 10	N = 9	N = 10	N = 51	N = 80
**Gender distribution**	60% f (6/10)	66.6% f (9/12)	50% f (5/10)	68.6% f (35/51)	65% f (52/80)
**Age mean (y) ± SD(y) [range(y)]**	10.6 ±4.06[4–16]	11.5 ± 4.3[5–18]	6.5 ± 3.1 [3–11]	9.9 ± 6[3y-18y]	9.8 ±4.25 [3–18]
**Self reported rhinitis**	10% Y(1/10)	0	0	23% Y(12/51)	16.2% Y(13/80)
**Smoking**	0	0	0	0	0
**Household income categories** *	80% A (8/10)	100% A (9/9)	90% A (9/10)	84.3% A (43/51)	86.2% A (69/80)
**SS16 result mean ± SD [range]**	14.4 ± 1.42[12–16]	14.4 ± 1.8[10–16]	11.9 ± 3.2 [7–16]	13.8 ± 2.4 [4–16]	13.7± 2.5 [4–16]
**PIT-SS16-Child resultmean ± SD [range]**	15.5 ±1.5[11–16]	15.7 ± 0.4[15–16]	14.0 ± 2.5 [9–16]	15.5±0.94 [12–16]	15.3± 1.3[9–16]

N = number, f = female, y = years, Y = yes, SS16 = 16 item Smell Identification Test; PIT-SS16-Child = picture identification test for the 16 items of the SS16, SD = standard deviation;

* = subjects were divided into 2 main categories of higher (A) or lower (B) income. The proportion of pre-school children in each pilot was as follows: pilot 1 = 1/10 (10.0%), pilot 2 = 1/9 (11.1%), pilot 3 = 4/10 (40%), pilot 4 = 9/51 (17.6%).

The final version of the test will be henceforth called SS16-Child. The SS16-Child was applied to 51 children aged 3 to 18 years in the final study. The final items and distractors used in the test are described in Table 2, in English. The images as well as the adapted Portuguese translation actually used for the SS16-Child in Brazil are provided as images in S1 Appendix and table in S2 Table). The raw data on all 51 children is provided as data in S3, S4 and S5 Tables), as well as for the 20 adults who performed the tests and were not included in the statistical analyses.

**Table 2:** Comparing the adult adaptation of the SS16 with the SS16-Child.

**Item number**	**Adults Brazilian version (from Silveira-Moriyama, 2008)**	**Child-friendly Brazilian version (SS16-Child)**
**1**	Strawberry, mulberry, orange*, pineapple	Cheese, olive, orange*, onion
**2**	Leather*, smoke, glue, grass	Leather*, milk, popcorn, banana
**3**	Cinnamon*, honey, chocolate, vanilla	Cinnamon*, French fries, fish, chocolate milk
**4**	Chives, pine scented disinfectant, mint*, onion	Water cracker, olive, mint flavoured candies*, onion
**5**	Coconut, banana*, walnut, cherry	Hamburger, banana*, barbecue, coffee
**6**	Peach, apple, orange, lemon*	Brazilian pasty, Brazilian baguette, popcorn, lemon*
**7**	Mint, liquorice*, cherry, cracker	Chocolate cake, fennel soap*, French fries, popcorn
**8**	Mustard, mint flavoured candy, rubber, solvent ink*	Barbecue, Brazilian Oreo, Brazilian cheese bread, paint*
**9**	Garlic*, onion, cabbage, carrot	Garlic*, Brazilian baby shampoo, apple, papaya
**10**	Cigarette, wine, smoke, coffee*	Brazilian pasty, papaya, gasoline, coffee*
**11**	Melon, orange, apple*, peach	Barbecue, garlic, apple*, fish
**12**	Cinnamon, clove*, pepper, mustard	Butter, clove*, Banana, guava
**13**	Pear, pineapple*, peach, plum	Butter, pineapple*, cheese, hamburger
**14**	Chamomile, raspberry, cherry, rose*	Lemon, Brazilian pasty, French fries, rose*
**15**	Honey, Brazilian alcoholic drink, anise*, pine scented disinfectant	Hamburger, chocolate cake, toothpaste*, cheese
**16**	Bread, cheese, ham, fish*	Strawberry, orange, rose, fish*

SS16-Child = 16 items of the Sniffin' Sticks smell test adapted to Brazilian children. All distractors are listed and the correct option is marked with *.

All children in the study scored 12 or more in the PIT-SS16-Child, and 37 (72.5%) scored 16. All items in this final version were correctly identified by 60% or more of the children tested (including pre-school children). Among children aged 10 or older, all items were recognized by at least 83.3% of children, and of the 20 adults included in the comparison group 18 scored 16 on the SS16-Child (and 2 scored 15). Some items were poorly recognized by children younger than 6 years old, despite the modifications. In this age group only 5 items (orange, banana, lemon, apple and pineapple) were recognized by more than 75% of subjects. Details of identification of items by age group are provided in the supplementary material (data in S6 Table).

**Fig 1:**
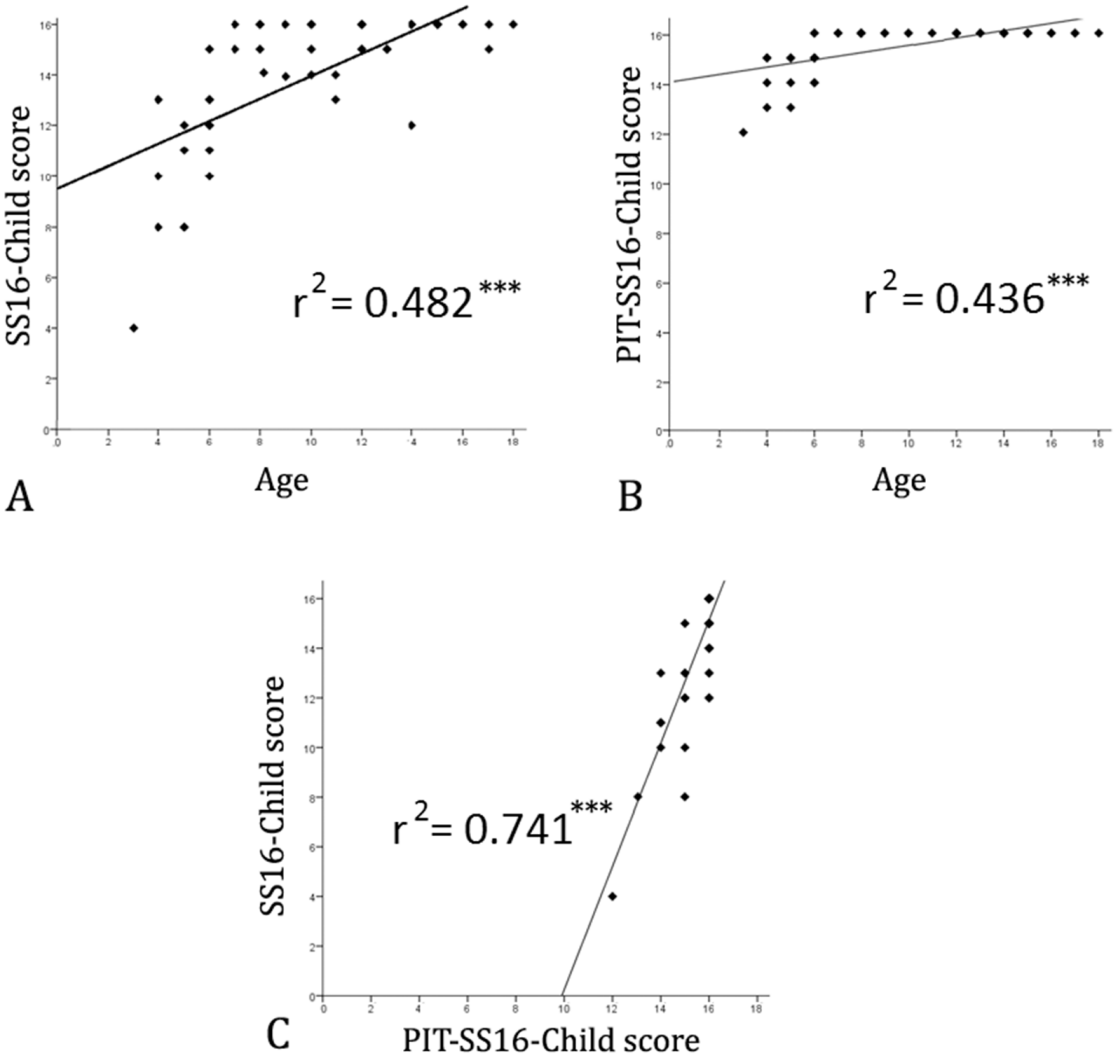
Correlations between SS16-Child, PIT-SS16-Child and age. SS16-Child: New version of Sniffin' Sticks smell test, adapted to Brazilian children; PIT-SS16-Child: Picture Identification Test, adapted for use with SS16-Child; *** highly significant association (p<0.001). A) r² = 0.482 indicating that the variation in age explains roughly 42% of the variation in the score of the test; B) r² = 0.436 indicating that 43% of the PIT-SS16-Child variation can be explained by the variation in age; C) This relationship explains roughly 74% of the variation in the SS16-Child data (r^2^ = 0.74) and is highly significant.

A strong correlation was found between age and SS16-Child score (r² = 0.482, p<0.001), age and PIT-SS16-Child score (r² = 0.436, p<0.001) and SS16-Child score and PIT-SS16-Child score (r² = 0.741, p<0.001). All these associations are represented in Fig 1. In addition, calculation of the partial correlation coefficients showed an independent effect of age on the SS16-Child when adjusted for PIT-SS16-Child (r^2^ = 0.109; p = 0.019) and of the PIT-SS16-Child on the SS16-Child when adjusted for age (r^2^ = 0.555, p<0.001).

For the same subjects a multiple linear regression was performed to determine the association between the SS16-Child and the independent variables of gender, household income and self-reported rhinitis, when adjusted for age and performance in the PIT. There were no evidence that these former variables influenced performance in the SS16-Child (p for gender = 0.647, p for income = 0.763, and p for self-reported rhinitis = 0.232). The multiple linear regression confirmed the independent effects of age (p = 0.024) and the PIT-SS16-Child score (p<0.001) on the olfactory performance (SS16-Child as dependent variable), with a larger effect size of the later (B for age = 0.17; 95% confidence interval = 0.024–0.317, and B for PIT-SS16-Child = 1.57; 95% confidence interval = 0.92–2.22).

## Discussion

This study shows that children as young as 5 could recognize most items from the SS16 when labels were translated and when necessary replaced by more culturally suitable items. It further demonstrates that in addition to age, which is known as a significant determinant of olfactory performance, cognition is an independent predictor of the results of the test and should be taken into consideration when interpreting test scores. Sniffin' Sticks is a commercially available smell test that has undergone extensive validation and that has been used internationally for more than a decade to diagnose hyposmia in adults. Therefore, any changes we made did not concern the smell themselves, which are produced by Burghart Messtechnik company (www.burghart-mt.de), but only the labels used in our adaptation, a procedure that can be reproduced in other countries in which a smell test is not available. The present study is the first to include the smell identification test in children outside of Europe, North-America or Australia and the results demonstrate that the cultural validation is an important step to adapt smell identification tests to children. Furthermore, the study shows that results in an adaptation (PIT-SS16-Child) of the PIT, a cognitive screening which was created to accompany the UPSIT test, is highly correlated with the performance in the SS16 when adapted to children, and therefore can be used to estimate the effect of cognition on the results of the olfactory task.

Although cognition is known to significantly impact smell identification performance in adults [22,30,31], no systematic study of the relationship between age, cognition and performance on smell identification has been included in most studies as can be seen from Table 3. Only Richman et al [2], Monnery-Patris et al [8], Dalton et al [9,10,13] and we have performed standardized cognitive screening. In addition, only our study and Richman et al [2] presented the statistical association between the olfactory and cognitive tests. Our study shows that a large part of the effect of age on olfactory performance is likely to be due to the effect of age on the cognitive abilities involved in the smell identification task (i.e., the same abilities which are involved in the PIT-SS16-Child) which include familiarity with the item, and also other abilities that impact identification and naming of figures, independently of familiarity with its smell and the capacity to form and identify olfactory imagery. The independent effect of the performance on the PIT-SS16-Child on the SS16 had a larger effect size than age itself, although both the multiple linear regression and partial correlation coefficient analyses showed a significant independent effect of age on olfaction, probably highlighting the role of olfactory learning that happens as children experiment various odours as they grow up [14]. This is visible in the data presented in Fig 1, which show that although most children older than 6 identified all pictures on the PIT-SS16-Child, many of these children failed to identify one or more odours in the SS16-Child. Although there is a possibility that these mistakes could be due to the odours presented not corresponding to the figures, this is unlikely given that 18 or the 20 adults included in the study correctly identified all 16 of the SS16Child items, indicating these items were highly identifiable. The item “leather”, for example, was correctly identified by 50/51 children in the PIT-SS16-Child, but it was only identified by 40/51 children in the SS16-Child, suggesting that although children knew what leather was, they probably were not familiar with its smell, or maybe were not able to form its olfactory image based on the odour provided, unlike adults which identified this item with more ease (19/20 identified correctly).

**Table 3:** Previous studies about smell identification test in children.

**PaperAuthor, year(Country)**	**SubjectsN (age in years)**	**Method**		**Olfaction associated with**			**Performance in pre-School Children**
		**Test Used**	**Cognitive Screening**	**Age**	**Gender**	**Cognition**	
**Richman, 1992 (USA)**	N = 175 (3.5–13)	Odorant Confusion Matrix	Peabody Picture Vocabulary Test	Yes	No	Yes	30/40 items poorly identified leading to proposal of a 5 item subscale for children <5
**Kobal, 2000 (USA)**	N = 24 (6–15)	Sniffin' Sticks	Not reported	Yes	No	Not studied	Not included
**Frank, 2003 (USA)**	N = 11 (3–9)	UPSIT	Not reported	Yes	Not reported	Not studied	Not described
**Frank, 2004 (USA)**	N = 39 (4–10)	UPSIT	Not reported	Yes	Not reported	Not studied	Not described
**Hummel, 2007(Germany)**	N = 111 (3–12)	Sniffin' Sticks	Not reported	Yes	No	Not studied	The data of children aged 3–5 years were not included (44% were incomplete)
**Laing, 2008 (Australia)**	N = 232 (5–7)	Not named	Not reported	Yes	No	Not studied	Reducing number of options and items improved performance in children aged 5–7y
**Monnery-Patris, 2009 (France)**	N = 152 (4–12)	Lyon Clinical Olfactory test	Evaluation of general verbal ability	Yes	Yes	Yes)	Six children aged 4y failed to understand the tasks.
**Dalton, 2009 (USA)**	N = 139 (3–17)	NIH Toolbox	Pictures recognition	Yes	Not reported	Not reported	Children under 5y performed poorly (average score 2.93 out of 6 items)
**Dalton, 2011 (USA)**	N = 419 (3–17)	NIH Toolbox	Picture recognition	Yes	Not reported	Not reported	Poor identification of 6/8 items by children aged 4y, and all 8 items by those aged 3y
**Dudova, 2011 (Czech Republic)**	N = 35 (6–18)	Sniffin' Sticks	Not reported	Yes	Not reported	Not studied	Not included
**Cameron, 2013 (USA)**	N = 152 (4–19)	Smell Wheel	Not reported	Yes	No	Not studied	Only 2/11 items recognized by ≥75% of children aged 4-5y
**Dalton, 2013 (USA)**	N = 3084 (3–18)	NIH Toolbox	Picture recognition	Yes	Not reported	Not reported	None of 6 items identified by ≥75% of children aged 3–5y
**Dzaman, 2013 (Poland)**	N = 85 (2.9–10)	Not named	Not reported	Yes	No	Not studied	Only 6/21 items identified by more of 75% children aged 2–4y
**Schriever, 2014 (Germany)**	N = 537 (6–17)	Sniffin' Sticks (Sniffin' Kids)	Not reported	Yes	No	Not studied	Not included
**Sorokowska, 2014 (Germany)**	N = 365 (4–15)	Sniffin' Sticks	Not reported	Yes	No	Not studied	Children aged 4 and 5 scored on average 11.5 and 10.7 out of 16 items (71.8% and 68.8%)
**Bastos, 2015 (Brazil)**	N = 51 (3–18)	Sniffin' Sticks (SS16-Child)	Modified Picture Identification Test	Yes	No	Yes	Only 5/16 items identified by ≥75% children aged 3–5y

The table shows only the results of smell identification tasks in control children (ie: subjects aged ≤18 years) extracted from the studies. Results for older subjects, patients with any given condition or other modalities of smell test are not presented. N = number of subjects, UPSIT = University of Pennsylvania Smell Identification Test, SS16-Child = adapted version of Sniffin' Sticks odour identification test used in this study.

Unlike Dalton et al [13] who performed the picture identification test before the olfactory task, as a means to exclude subjects unable to perform, and also clarify the meaning of images to smaller children before they performed the test, we only offered the PIT-SS16-Child after children completed the SS16-Child. Therefore children’s previous cognitive experiences with the items and their pre-test capacity to associate the images with the items were more influential in our test. Dalton did not report the statistical association between performance in the picture and olfactory identification tasks.

Our findings largely reproduce other common findings in the studies included in Table 3 [2–10,12,13,15–17], showing that for any given olfactory test, the effect of age on olfaction will eventually reach a ceiling and children will no longer perform differently from young adults. It is predictable that tests that used more highly identifiable items like ours tended to level down the results in different ages to the same common denominator, generating an earlier ceiling effect (ie: older teenagers identified almost all items, please see the data in S6 Table). We avoided direct comparisons with adults because the limited sample size using a low sensitivity test can generate non-significant results for certain age groups, without actually reflecting lack of subtle differences between the groups. A future study, comparing the performance of teenagers and adults in an adult test (therefore a more sensitive instrument) would be more suited to fulfil this specific knowledge gap. To enable other researchers to compare the data from the children and adults in our study, we have included the data from the adults in the S7, S8 and S9 Tables). The development of cognition and olfaction in children mirrors the olfactory decline and cognitive loss observed in the elderly [38], and most studies that included wide age groups show that olfactory performance peaks in young adults. Although some variation can be seen in the SS16-Child result up to 18 years, the largest variation is found amongst children aged 10 or less. From our data, and the other data existing in the literature, it looks like olfactory learning is much more significant in pre-school children. Children younger than 3 are unlikely to perform well on the smell identification test of the SS16-Child, and children older than 10 are likely to score correctly on all or almost all 16 items if they have normal olfaction. This probably makes the SS16-Child in its current form a test with a high specificity for hyposmia in teenagers (ie: if an adolescent looses a couple of points, we can conclude there is a high chance of a true smell deficit), but at the cost of sensitivity (ie: because the smell identification test is very “easy” it is possible that subjects with only mild smell deficit could still perform very well in the test). But this larger “reserve” might be useful to test olfaction in conditions which cause cognitive deficit, because children might still be able to perform the test even with some cognitive loss, making this test more likely to be suitable in these conditions. This needs to be studied further in a study involving children with confirmed smell disorders, and also children with neurological and cognitive conditions.

In ours and in most studies, pre-school children seem to form a separate group when it comes to olfactory identification. Children younger than 6 presented considerable underperformance compared to older children in ours and other studies [2,6,8–10,12–14,16], suggesting that separate subscales, or separate norms for the same scales should be applied to them. Most authors found only up to 5 or 6 of the items tested in any test were suitable for this age group. In our study only fruit items (orange, banana, lemon, apple and pineapple) were highly identified (more than 75%) by children younger than 6. It might be that further development of tests specifically targeting this age group might provide a longer test with high identifiability. Even young children were able to perform the PIT-SS16-Child after the SS16-Child with good engagement scoring at least 12 points, showing they are amenable to longer tests and that the length of the SS16-Child was not a limitation to their performance.

With cultural adaptations of the distractors and using figures to represent distractors and items, the SS16 could be used cross-culturally to test Brazilian children. The performance of children in our study was similar to the recent study performed in Germany [15] where the SS16 was developed, with the difference that while in Germany children miss-identified the item “apple” and "turpentine" which was excluded from the so-called “Sniffin-Kids”, in Brazil children did not recognize “cinnamon”, raising the point that in further cultural adaptations for other countries, the 16 item test might be more useful than adapting the Sniffin-Kids directly.

Even though the current study included a limited number of subjects, it suggests that the SS16-Child could be used cross-culturally to test olfaction in children older than 5, and that a subscale could be used in younger children. The current version of the SS16-Child when applied to Brazilian children might render the test less sensitive to smell deficit in older children. The results suggests that the PIT-SS16-Child could be used as a screening test for cognitive abilities necessary to perform the test, and as an adjustment method for the effects of cognitive abilities on olfaction when testing smaller children or patients with potential cognitive deficits. In fact, given that the effect size of the PIT-SS16-Child was larger than the effect size of age, this adjustment might be paramount for the understanding of the results of the test. Larger studies including cognitive and olfactory testing in children from all age groups are warranted.

The creation of truly “cross-cultural” smell identification tests suitable to most major cultures worldwide would enable multicenter clinical studies of olfaction and has been target of clinical research [39]. However the current study only involved Brazilian children, precluding direct conclusions regarding suitability of the SS16 for use in children of other backgrounds. It is possible that a similar process of adaptation with replacement of labels and the application of adapted picture identification tests could be performed successfully in other cultures, but further studies are warranted. The SS16 is a clinical test of olfaction used to detect clinically meaningful hyposmia and consequently guide the clinical management of the patients. The values used as cut-off for hyposmia are usually arbitrarily defined by the 10th percentile [3,7,15,16,29] of a large sample of subjects of a given age group. Therefore, in order to provide cut-off values for this test, studies in larger cohorts are warranted. The main contributions of this work are (1) to demonstrate that the original version of the Sniffin Sticks can be applied to Brazilian children, (2) to indicate that future studies in larger cohorts aiming to determine cut-off values for the SS16 in children (either in Brazil or other countries) should include cognitive testing to aid interpretation of the results correctly and that (3) studies using other smell testing kits may benefit from the inclusion of some form of cognitive testing to aid interpretation of the test.

## Supporting Information

S1 Appendix SS16-Child images. (PDF)

S1 Table Changes in each pilot. * = Correct alternatives for items are marked by a star.
(DOCX)

S2 Table Portuguese version of SS16-Child. *Correct items.
(DOCX)

S3 Table Demographic data of 51 children participating in the final study. Legend: f—female; m = male; A—family income less than 5 Brazilian minimum wage; B—family income over than 5 Brazilian minimum wage.
(DOCX)

S4 Table SS16-Child scores of 51 children participating in the final study. Legend: 1—correct answer; 0—wrong answer.
(DOCX)

S5 Table PIT-SS16-Child scores of 51 children participating in the final study. Legend: 1—correct answer; 0—wrong answer.
(DOCX)

S6 Table Percentage of correct identification by age group. The columns show the percentage of subjects in each age group who correctly identified each item. Cells are coloured according to the following rules: ≥75% green, 60–74.9% yellow, <60% red. N = number of subjects; y = years.
(DOCX)

S7 Table Demographic data of adults. Legend: f—female; m = male; A—family income less than 5 Brazilian minimum wage; B—family income over than 5 Brazilian minimum wage.
(DOCX)

S8 Table Adults results in SS16-Child. Legend: 1—correct answer; 0—wrong answer.
(DOCX)

S9 Table Adults results in PIT-SS16-Child. Legend: 1—correct answer; 0—wrong answer.
(DOCX)
