# Supplementary material for: Effects of Age and Cognition on a Cross-Cultural Paediatric Adaptation of the Sniffin' Sticks Identification Test
Source: PLoS One. 2015 Aug 12;10(8):e0131641. doi: 10.1371/journal.pone.0131641 (PMC4534354; doi:10.1371/journal.pone.0131641)
Supplement: S1 Appendix — (PDF) [file pone.0131641.s001.pdf]

A

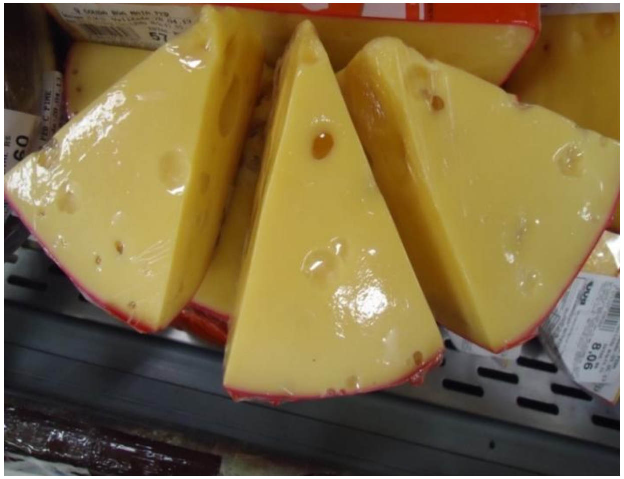

CHEESE

B

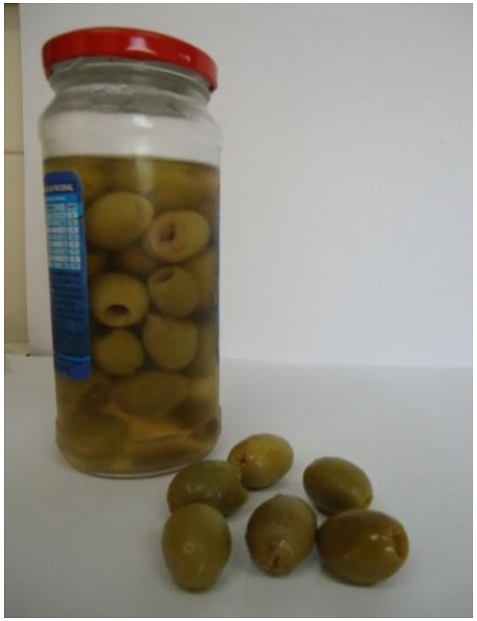

OLIVE

C

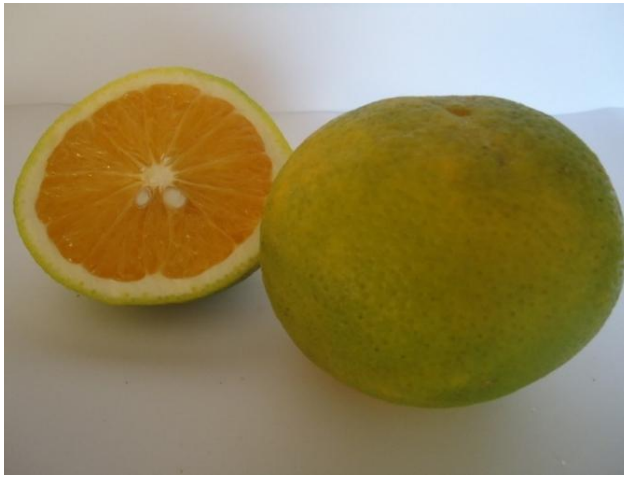

ORANGE

D

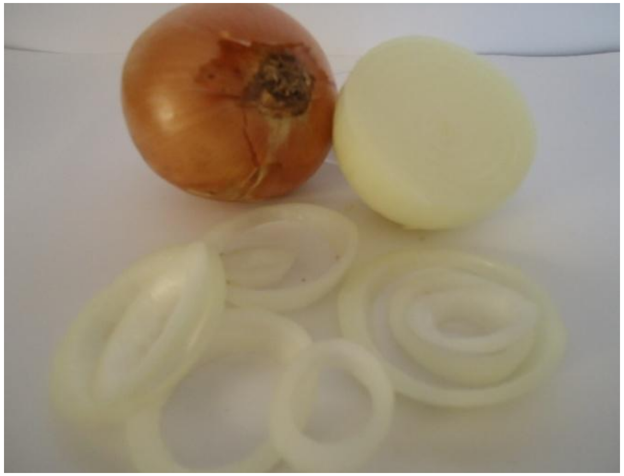

ONION

A

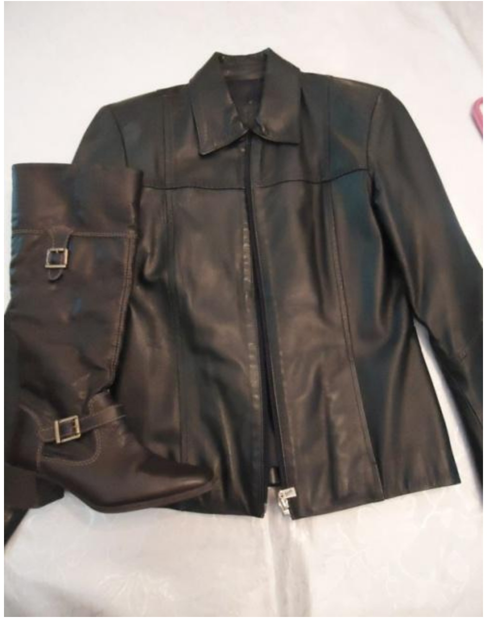

LEATHER

B

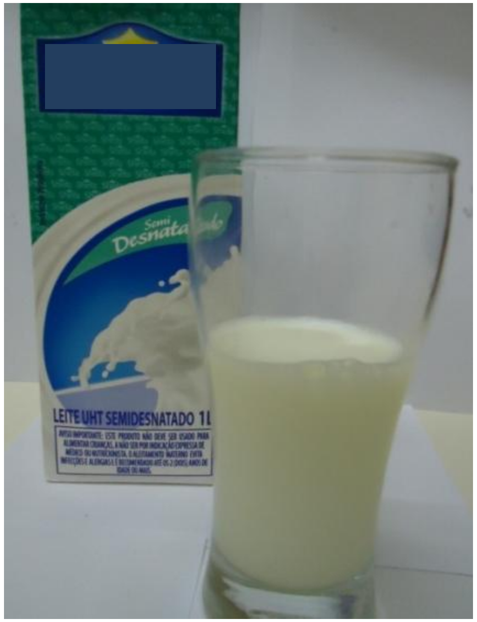

MILK

C

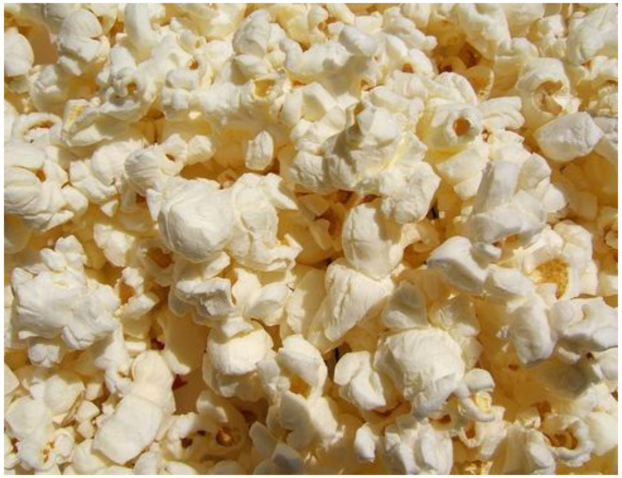

POPCORN

D

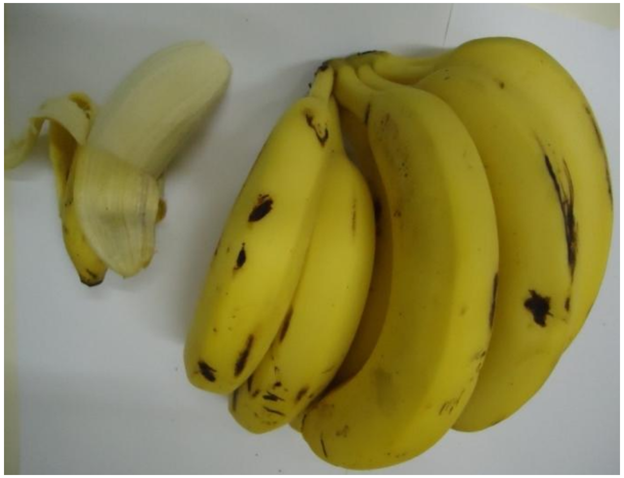

BANANA

A

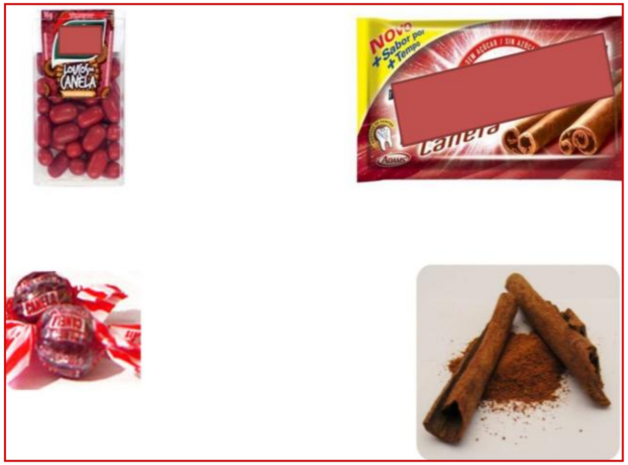

CINNAMON

B

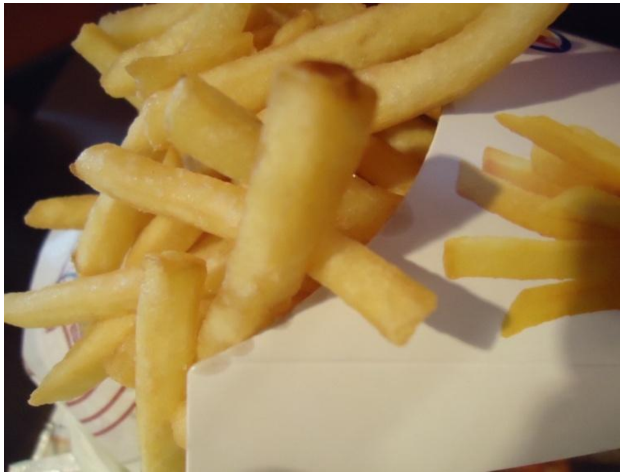

FRENCH FRIES

C

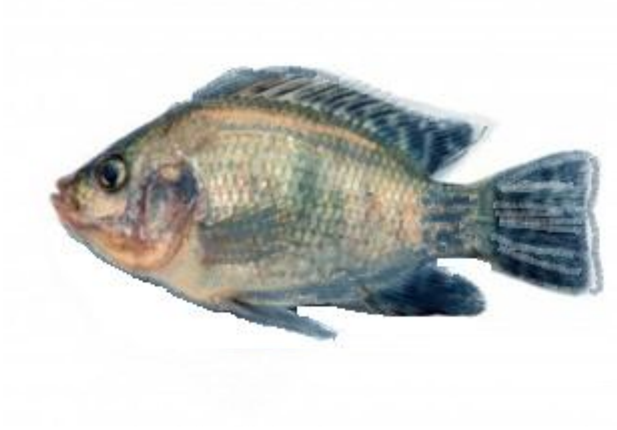

FISH

D

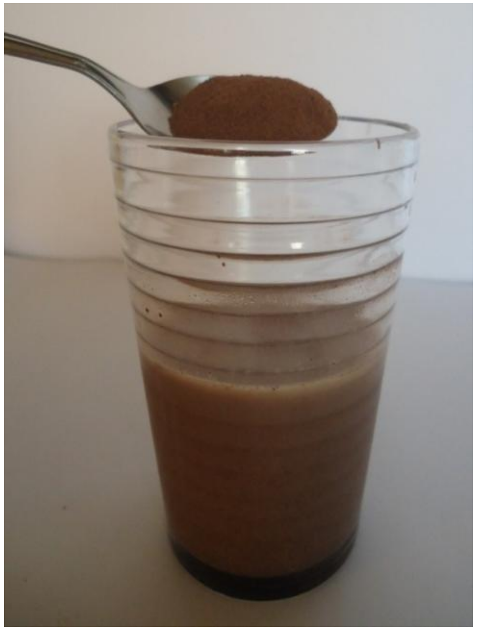

CHOCOLATE MILK

A

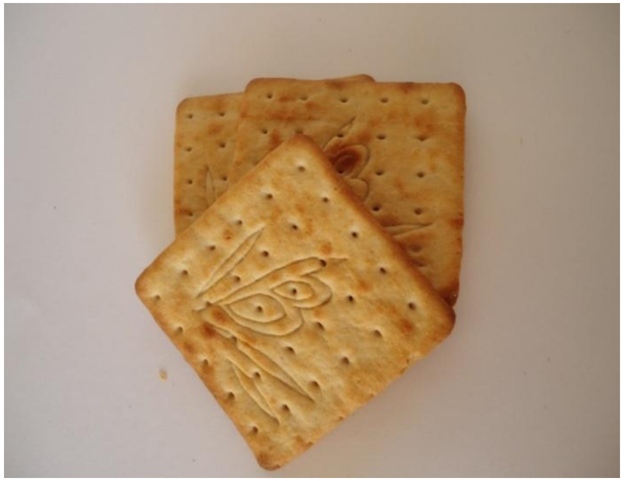

CRACKER

B

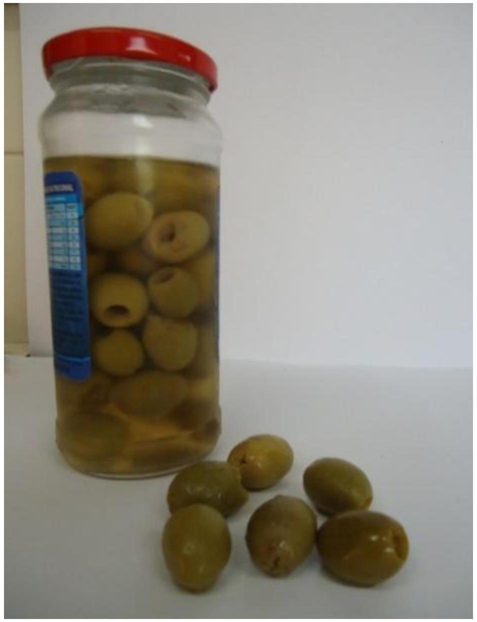

OLIVE

C

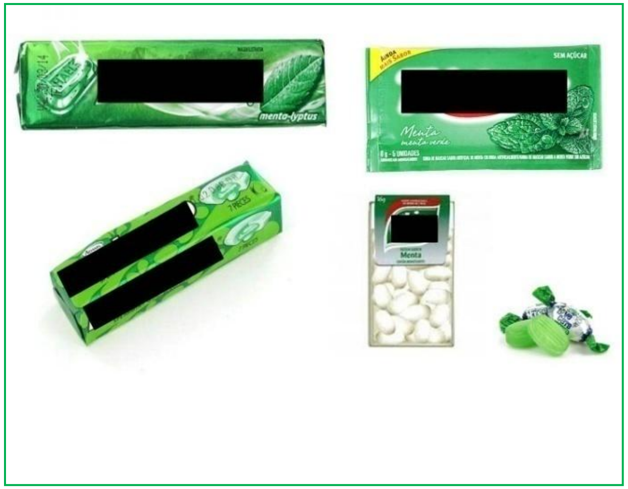

MINT

D

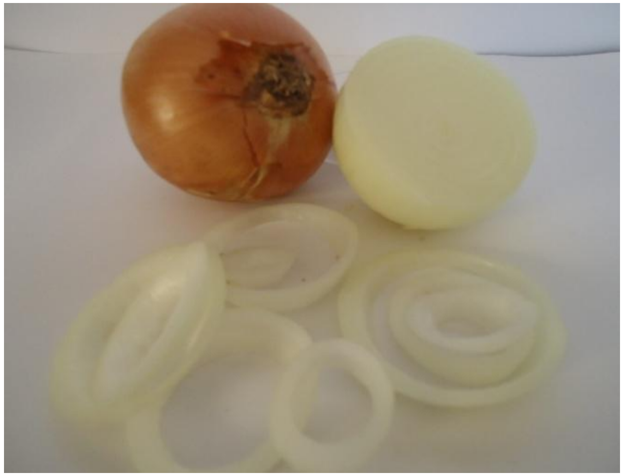

ONION

A

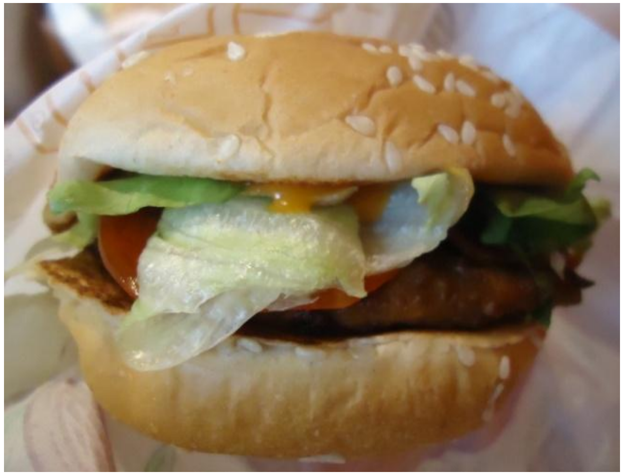

HAMBURGUER

B

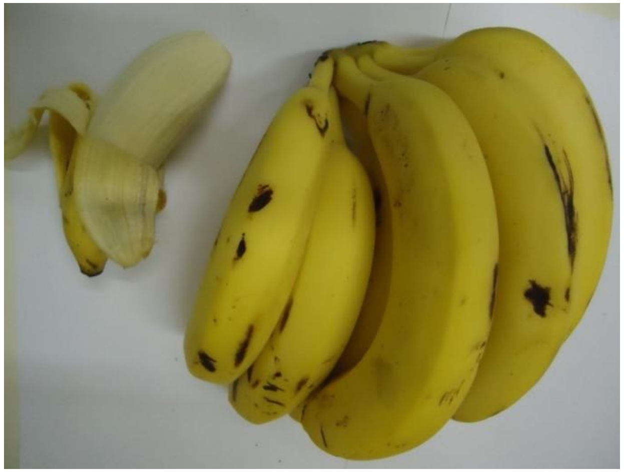

BANANA

C

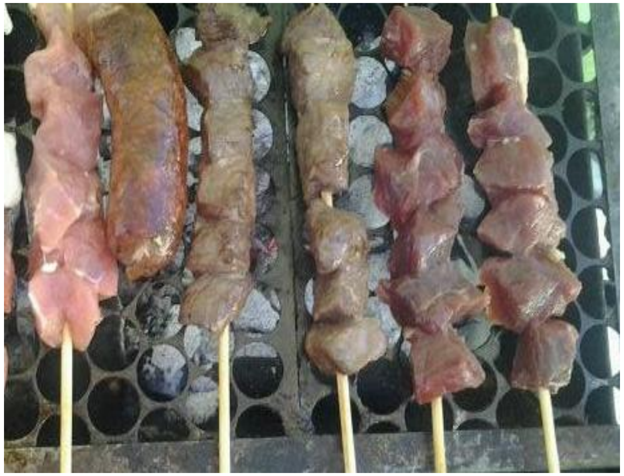

BARBECUE

D

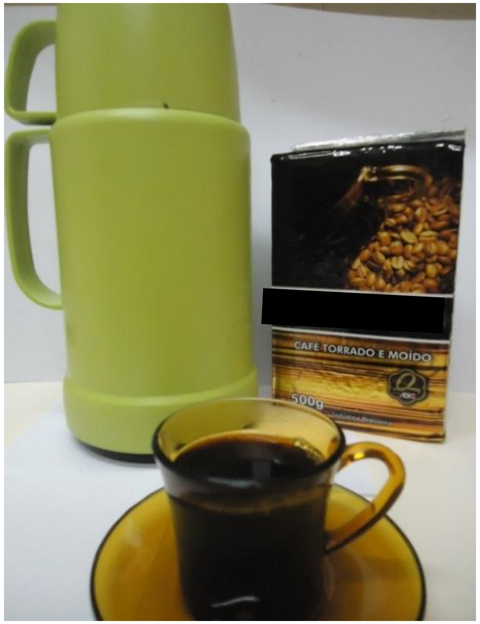

COFFEE

A

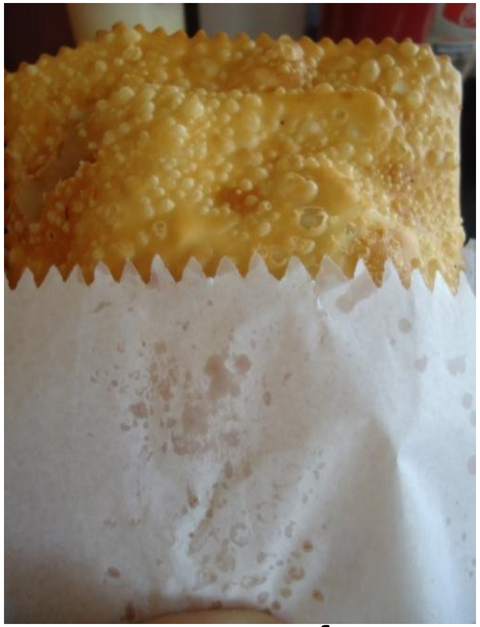

BRAZILIAN ‘PASTEL’

B

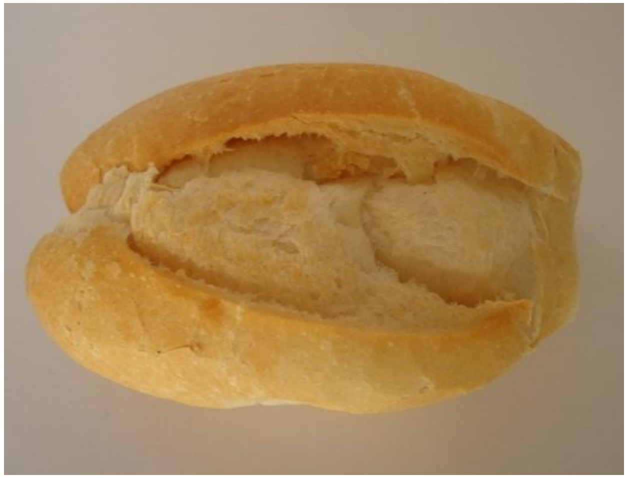

BREAD

C

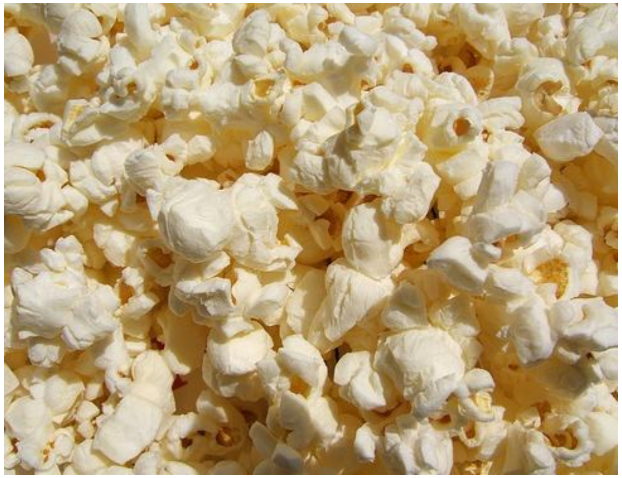

POPCORN

D

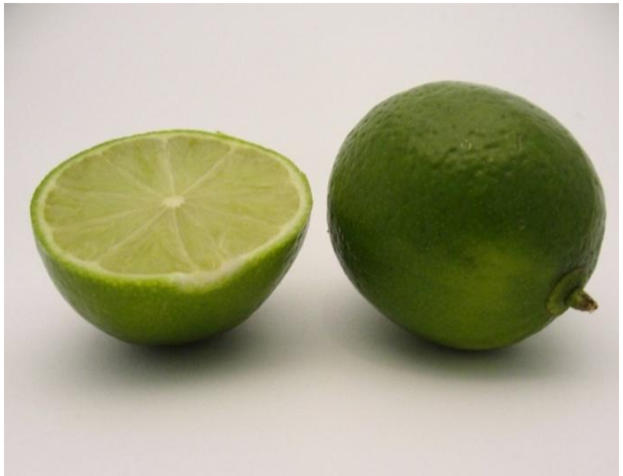

LEMON

A

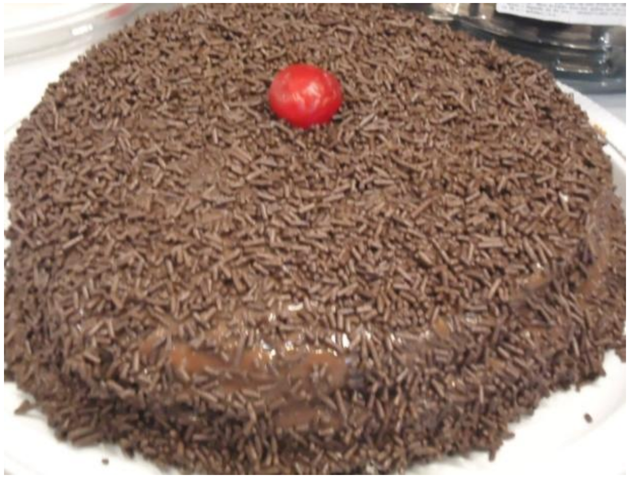

CHOCOLATE CAKE

B

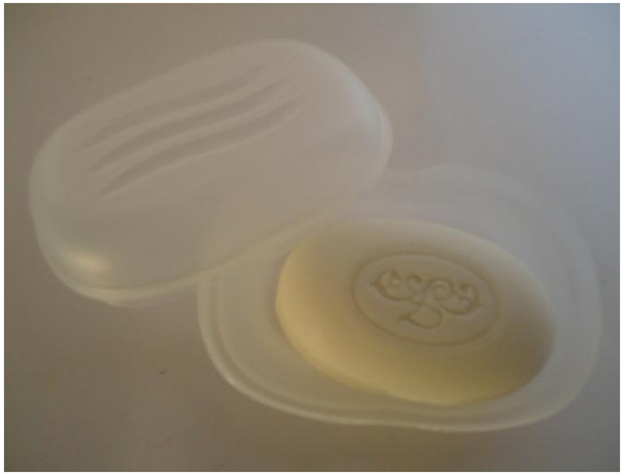

FENNEL SOAP

C

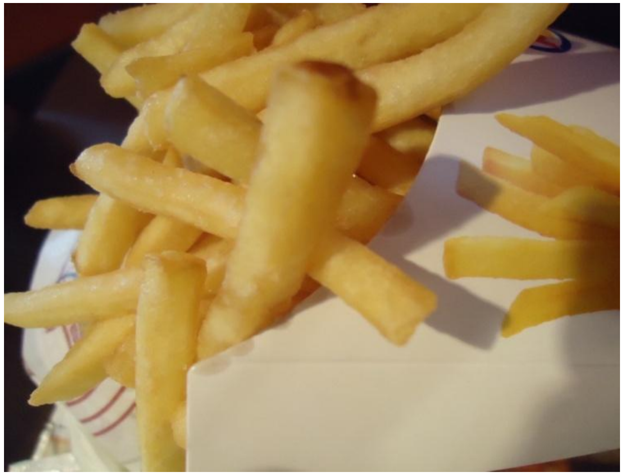

FRENCH FRIES

D

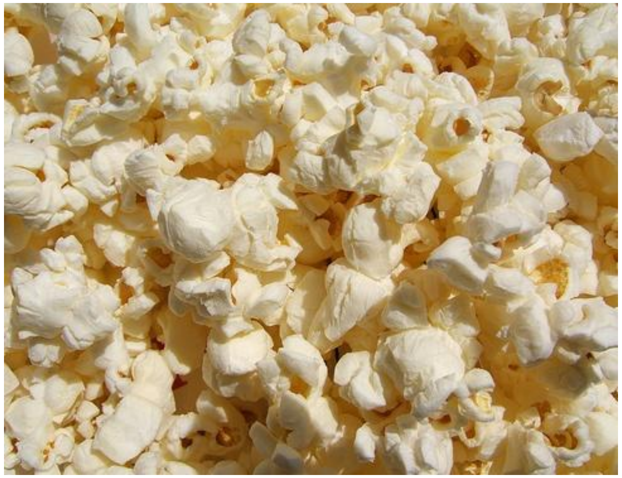

POPCORN

A

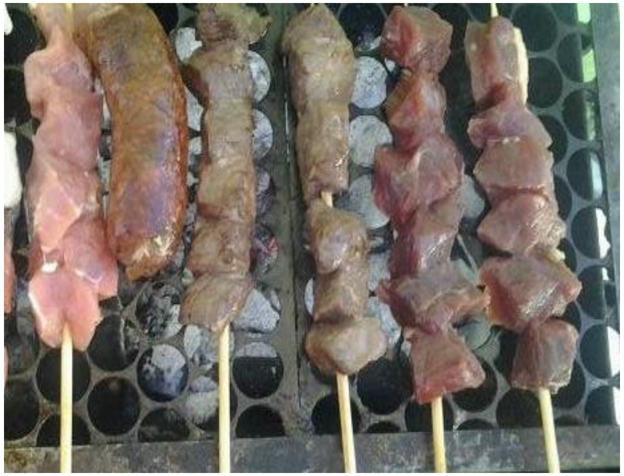

BARBECUE

B

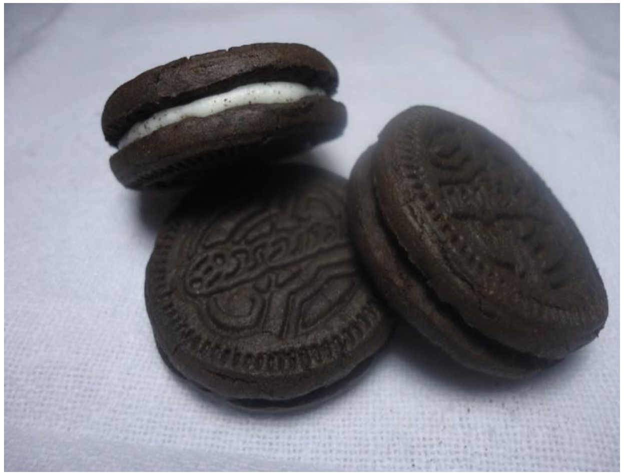

BRAZILIAN OREO

C

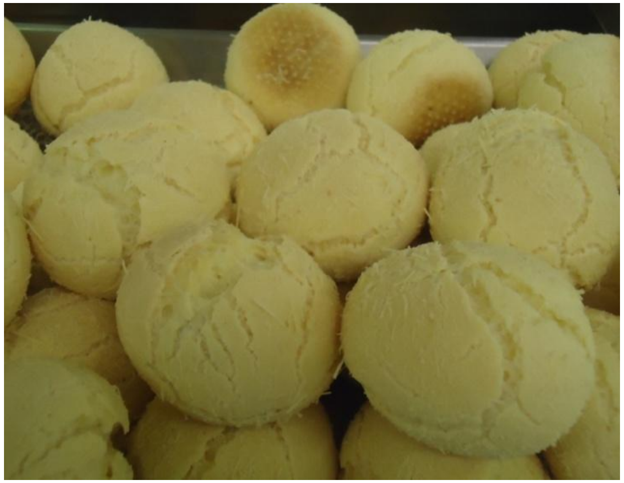

BRAZILIAN CHEESE BREAD

D

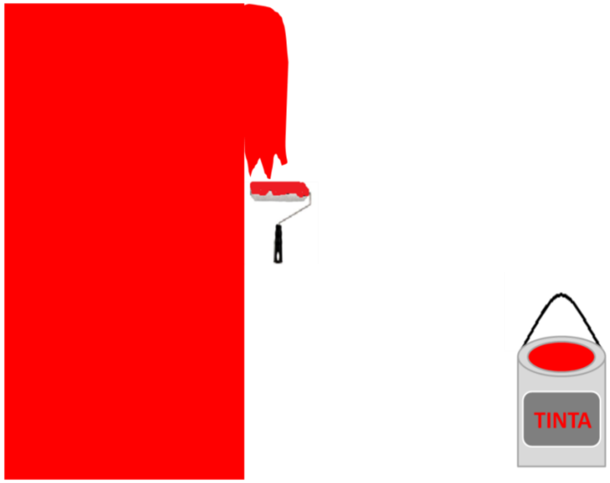

PAINT

A

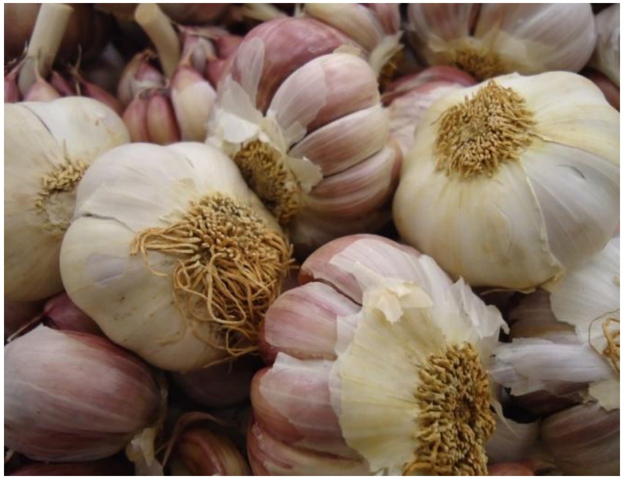

GARLIC

B

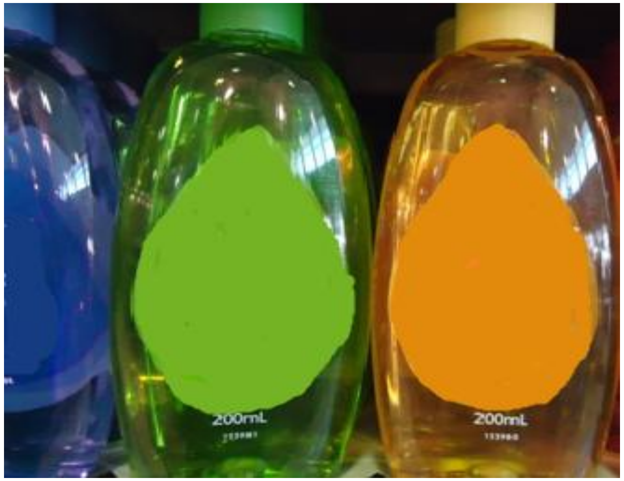

SHAMPOO

C

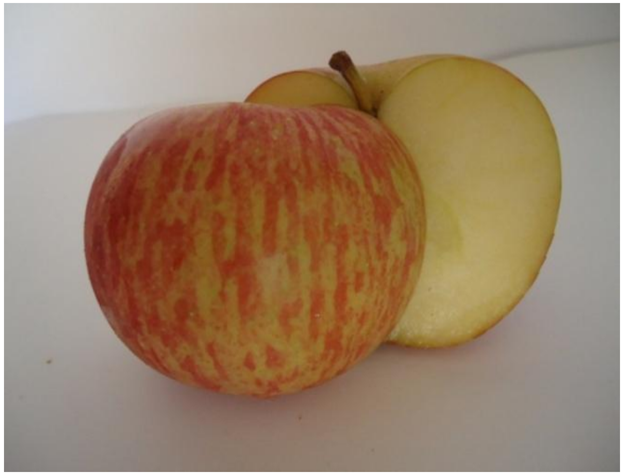

APPLE

D

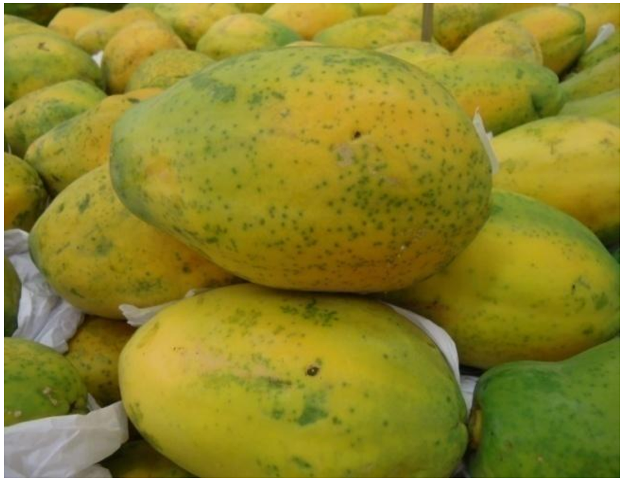

PAPAYA

A

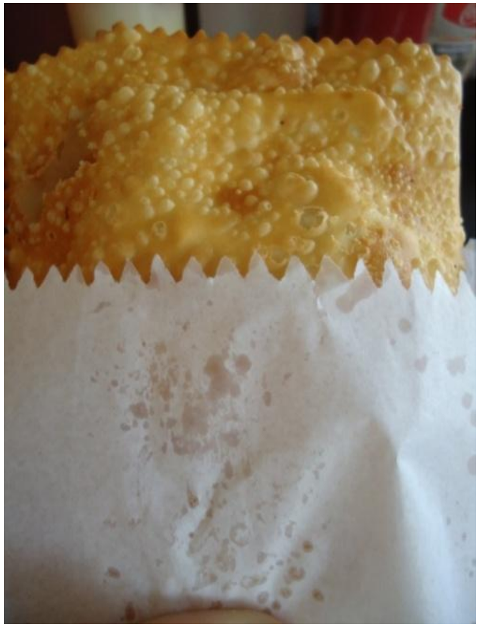

BRAZILIAN ‘PASTEL’

B

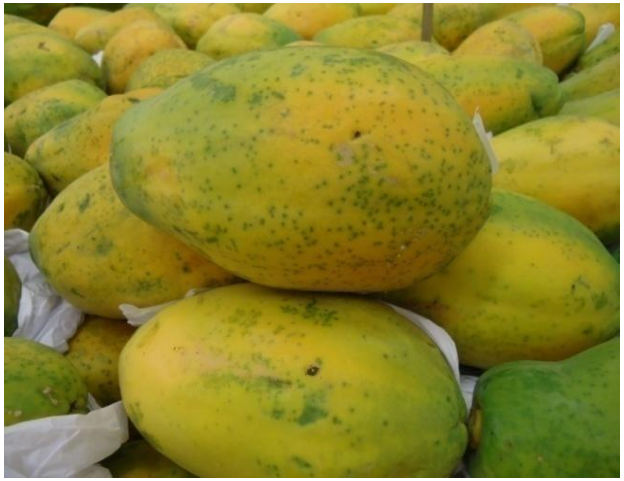

PAPAYA

C

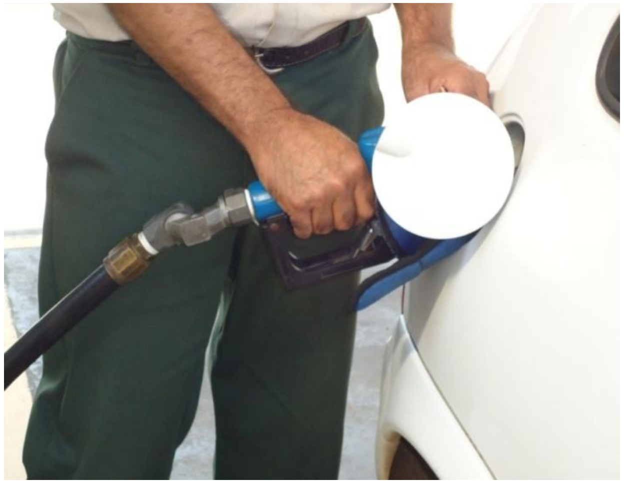

GASOLINE

D

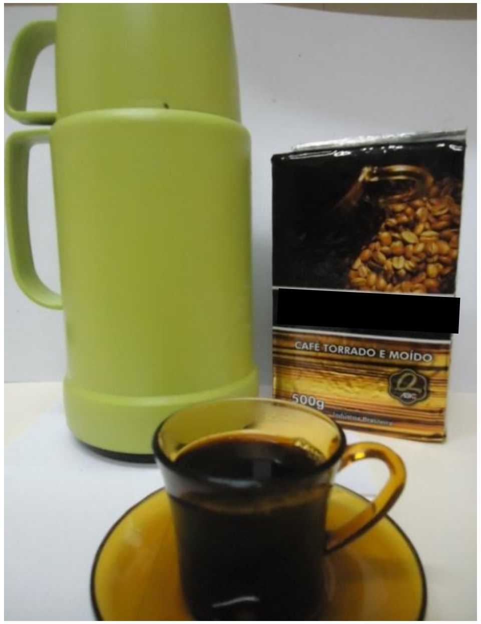

COFFEE

A

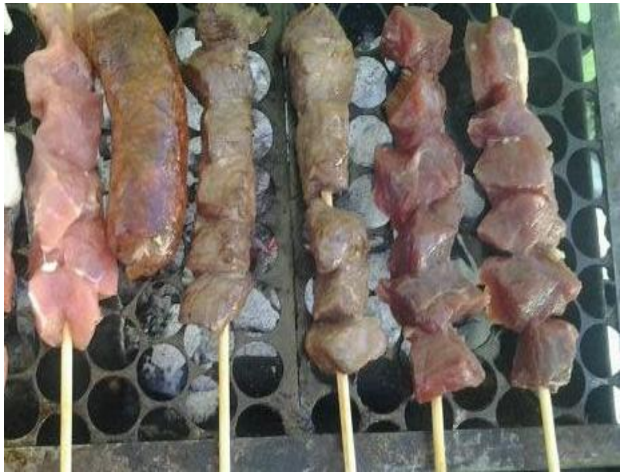

BARBECUE

B

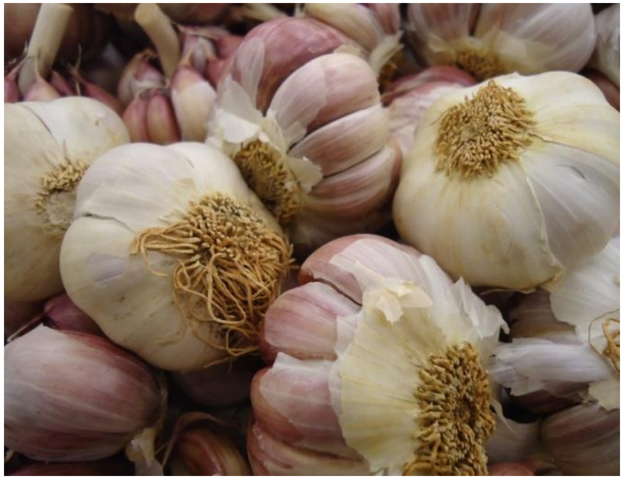

GARLIC

C

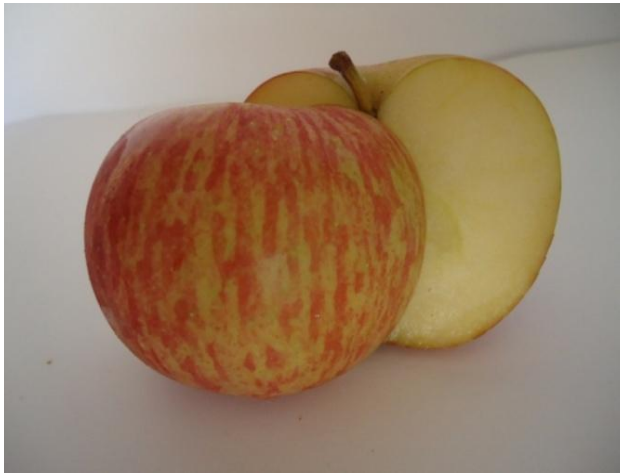

APPLE

D

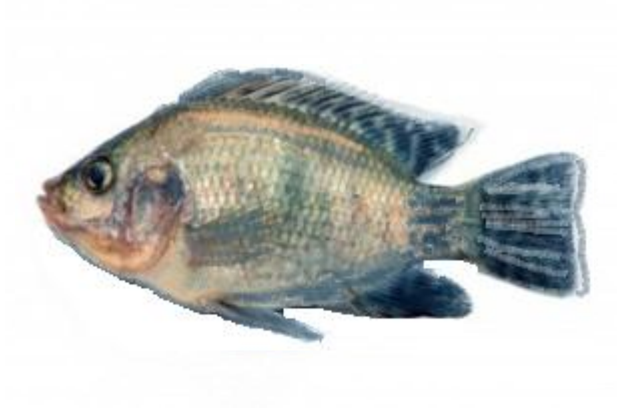

FISH

A

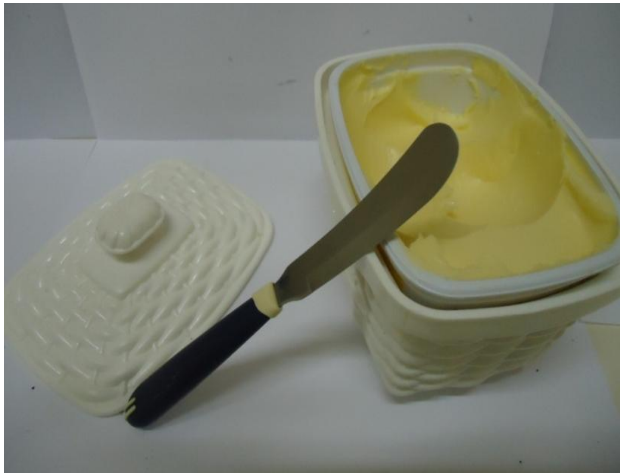

BUTTER

B

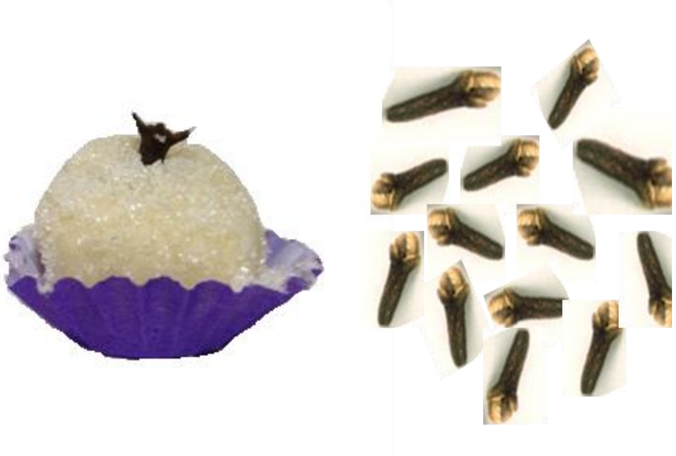

CLOVE

C

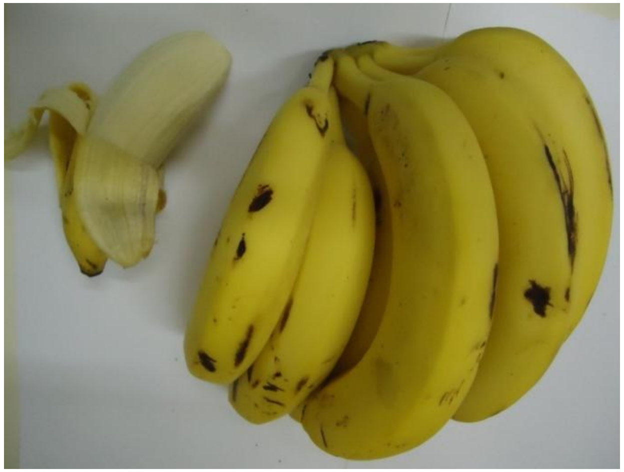

BANANA

D

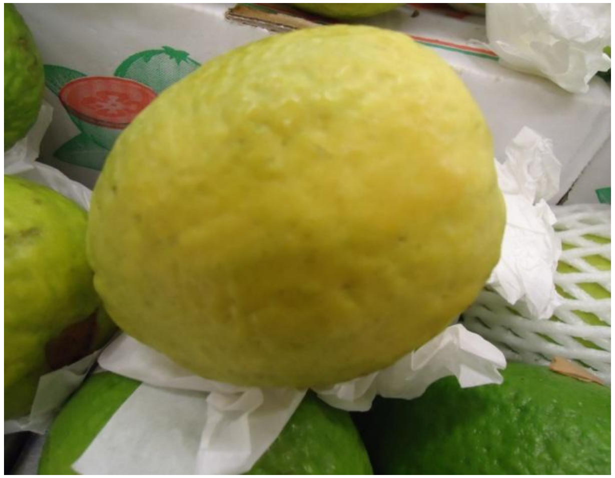

GUAVA

A

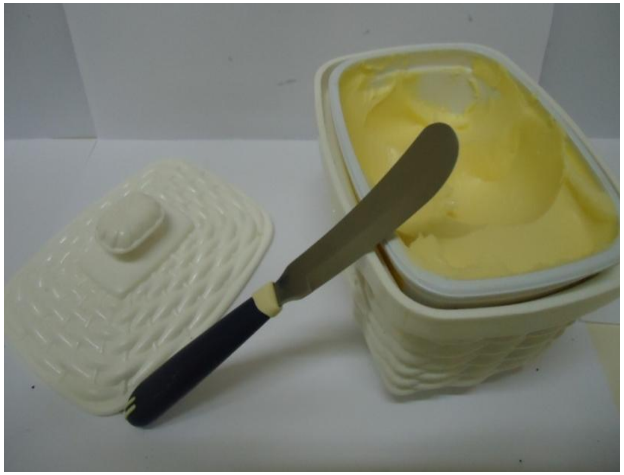

BUTTER

B

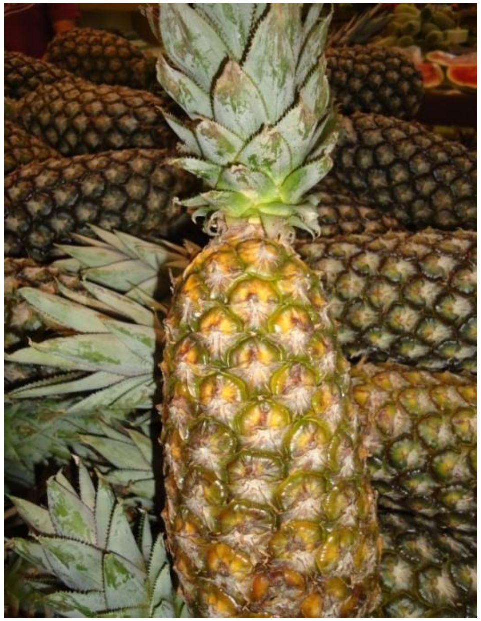

PINEAPPLE

C

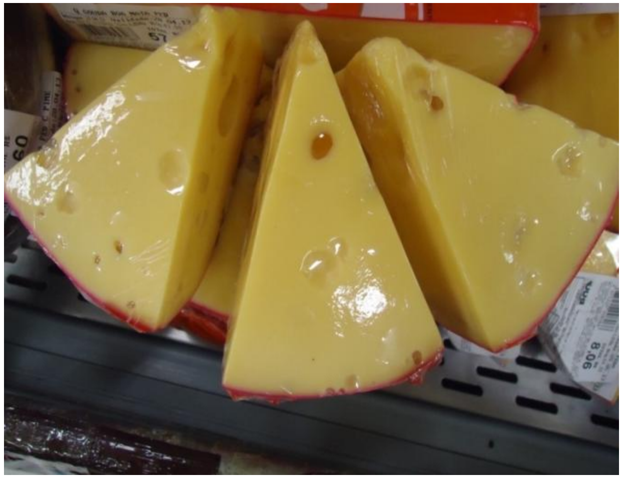

CHEESE

D

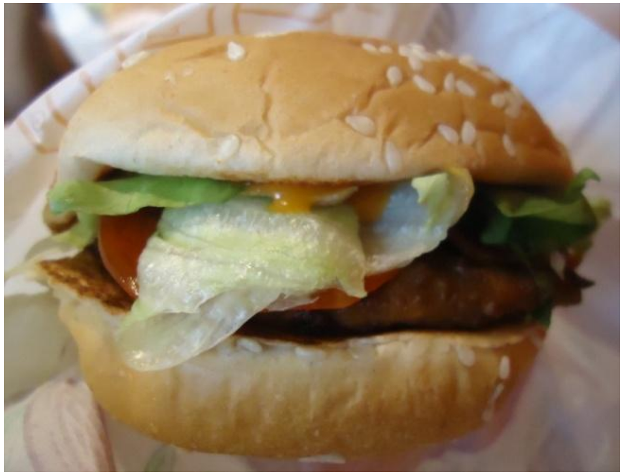

HAMBURGUER

A

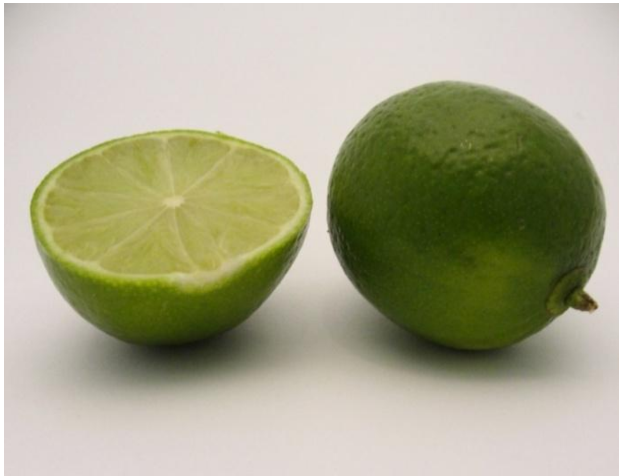

LEMON

B

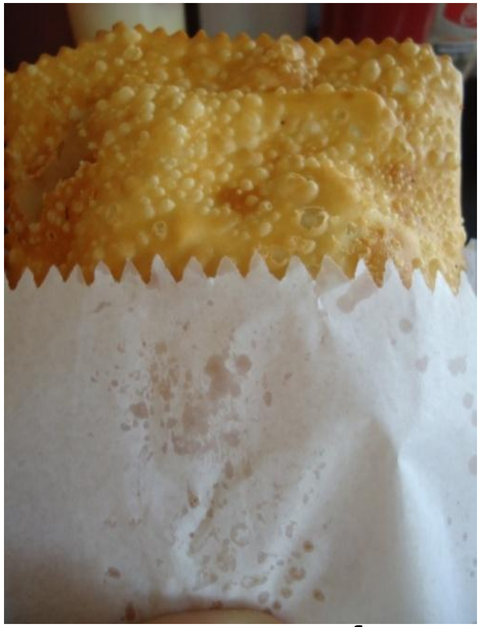

BRAZILIAN ‘PASTEL’

C

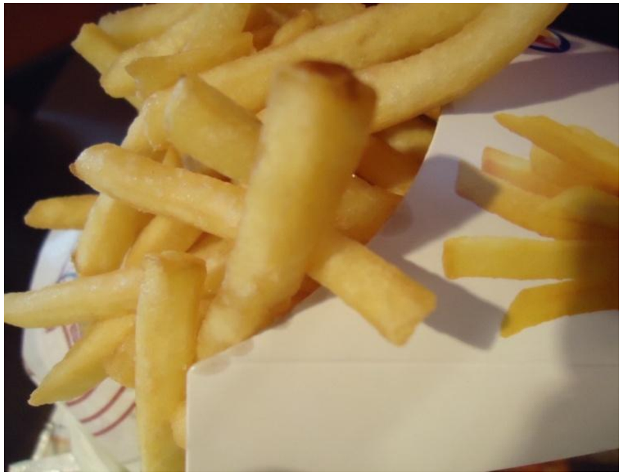

FRENCH FRIES

D

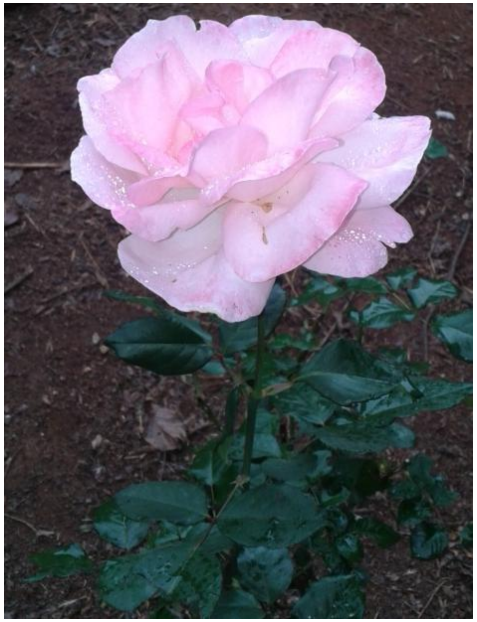

ROSE

A

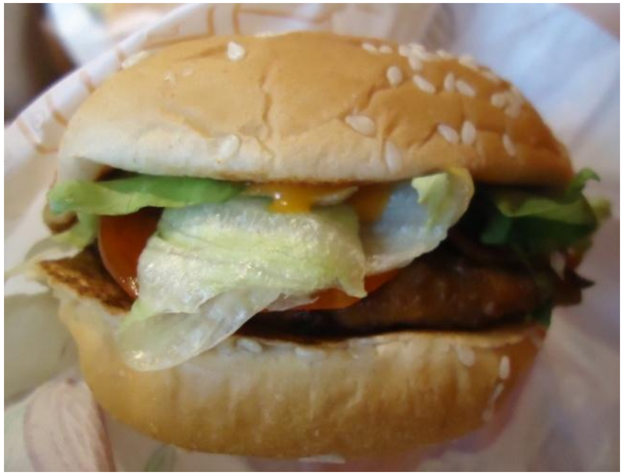

HAMBURGUER

B

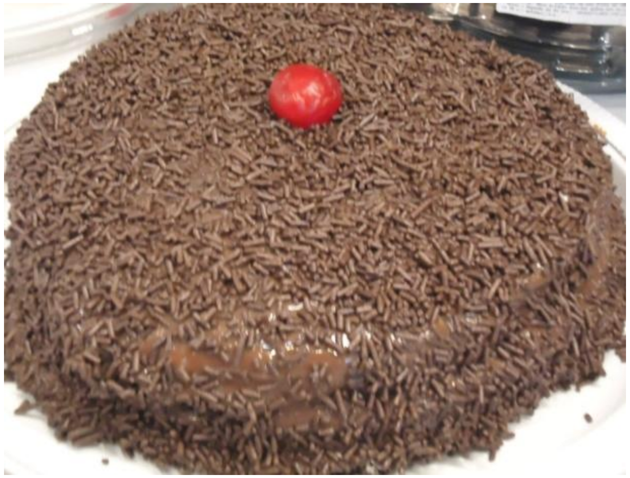

CHOCOLATE CAKE

C

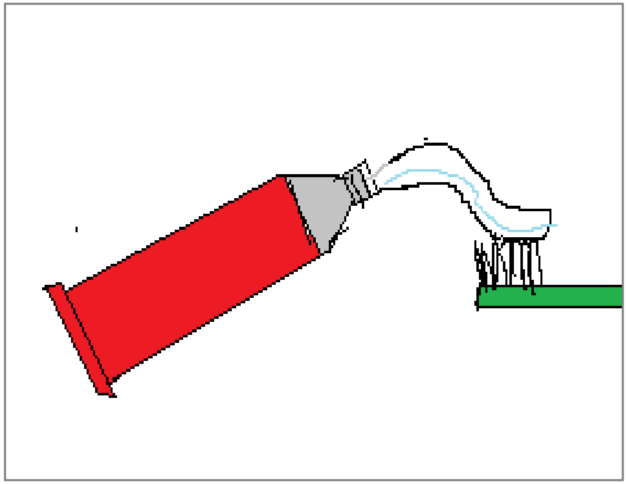

TOOTHPASTE

D

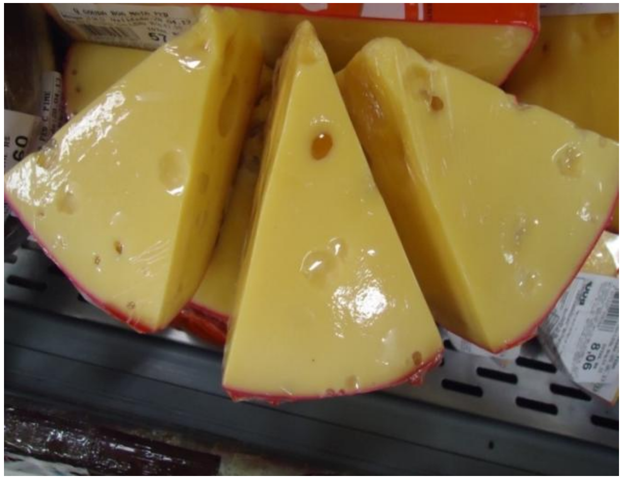

CHEESE

A

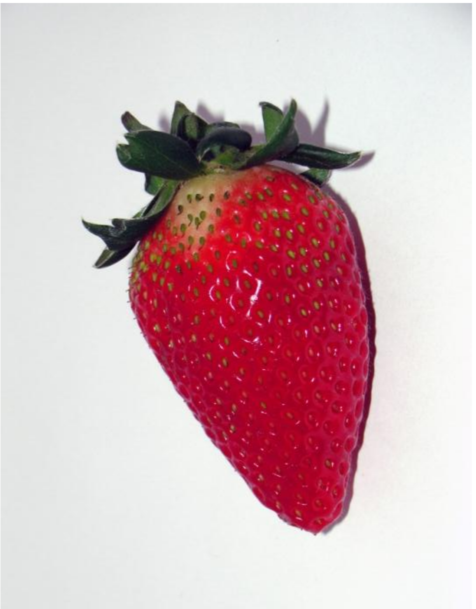

STRAWBERRY

B

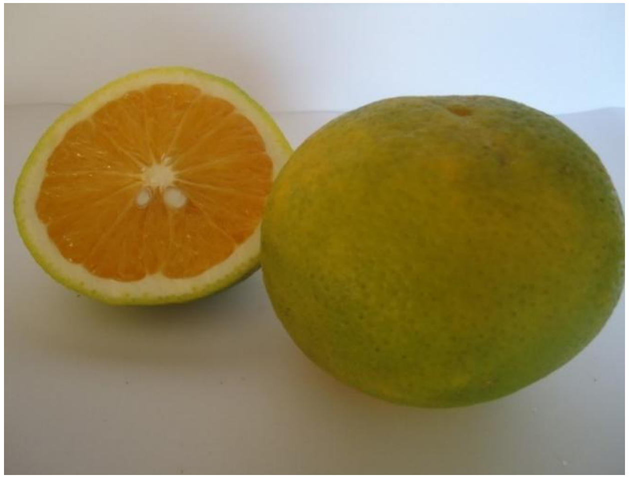

ORANGE

C

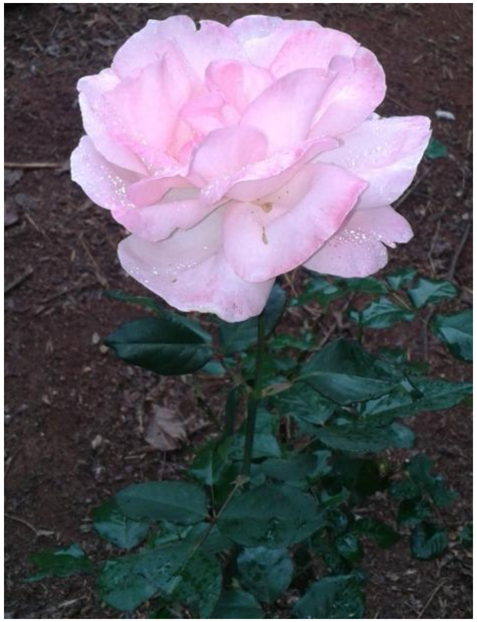

ROSE

D

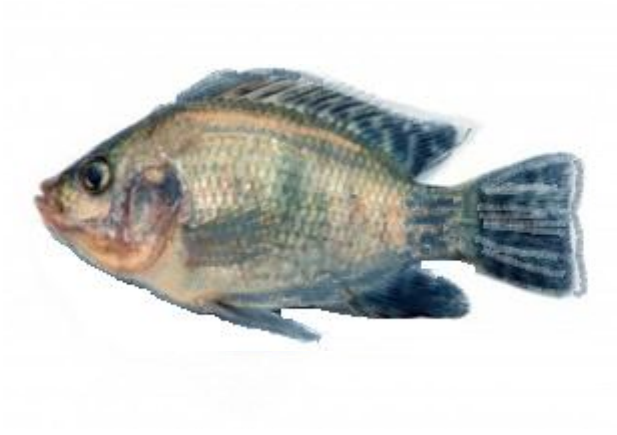

FISH
