# Supplementary material for: Effects of Age and Cognition on a Cross-Cultural Paediatric Adaptation of the Sniffin' Sticks Identification Test
Source: PLoS One. 2015 Aug 12;10(8):e0131641. doi: 10.1371/journal.pone.0131641 (PMC4534354; doi:10.1371/journal.pone.0131641)
Supplement: S1 Table — * = Correct alternatives for items are marked by a star. (DOCX) [file pone.0131641.s002.docx]

**S1 Table - Changes in each pilot**

To enable international readers to understand the adaptation process we have presented here free translations of the words used in the adapted translation. Please note some of the items which were used due to its familiarity in Brazil, do not have direct correspondents in the English language, and therefore we have used words that represent approximate concepts. This is not intended to detail the process of adaptation for this specific test, but rather to give researchers an approximate idea of the amount of changes needed in this originally German test, so we could achieve high rates of identifiability amongst children of various age groups tested. The tables also demonstrate that many distractors which were originally non-contrasting (like item one, which originally had various fruits as options) were replaced by more contrasting options.

| PILOT 1 – Please not almost all items suffered significant changes from the adult to the paediatric version | | |
| --- | --- | --- |
| Item number | **Adults Brazilian version (from Silveira-Moriyama, 2008)** | **Pilot 1** |
| 1 | Strawberry, mulberry, orange*, pineapple | Cheese, olive, orange*, onion |
| 2 | Leather*, smoke, glue, grass | Leather*, milk, chocolate cake, popcorn |
| 3 | Cinnamon*, honey, chocolate, vanilla | Cinnamon*, French fries, fish, chocolate milk |
| 4 | Chives, pine scented disinfectant, mint*, onion | Water cracker, olive, mint flavoured candy*, onion |
| 5 | Coconut, banana*, walnut, cherry | Hamburger, banana*, barbecue, coffee |
| 6 | Peach, apple, orange, lemon* | Brazilian pasty, Brazilian baguette, popcorn, lemon* |
| 7 | Mint, liquorice*, cherry, cracker | Chocolate cake, fennel soap*, French fries, popcorn |
| 8 | Mustard, mint flavoured candy, rubber, solvent ink* | Tangerine, orange, Brazilian cheese bread, paint* |
| 9 | Garlic*, onion, cabbage, carrot | Garlic*, Brazilian baby shampoo, apple, papaya |
| 10 | Cigarette, wine, smoke, coffee* | Brazilian pasty, papaya, gasoline, coffee* |
| 11 | Melon, orange, apple*, peach | Barbecue, chocolate milk, apple*, fish |
| 12 | Cinnamon, clove*, pepper, mustard | Butter, clove*, Brazilian baguette, guava |
| 13 | Pear, pineapple*, peach, plum | Chocolate milk, pineapple*, cheese, hamburger |
| 14 | Chamomile, raspberry, cherry, rose* | Lemon, Brazilian pasty, French fries, rose* |
| 15 | Honey, Brazilian alcoholic drink, anise*, pine scented disinfectant | Brazilian baguette, tangerine, toothpaste*, banana |
| 16 | Bread, cheese, ham, fish* | Strawberry, watermelon, rose, fish* |
| PILOT 2 – here only 3 items were changed as discriminated below | | |
| Item number | **Pilot 1** | **Pilot 2** |
| 8 | Tangerine, orange, Brazilian cheese bread, paint* | Barbecue, Brazilian Oreo, Brazilian cheese bread, paint* |
| 11 | Barbecue, chocolate milk, apple*, fish | Barbecue, garlic, apple*, fish |
| 15 | Brazilian baguette, tangerine, toothpaste*, banana | Brazilian baguette, olive, toothpaste*, cheese |
| PILOT 3 - here only 2 items were changed as discriminated below | | |
| Item number | **Pilot 2** | **Pilot 3** |
| 15 | Brazilian baguette, olive, toothpaste*, cheese | Hamburger, chocolate cake, toothpaste*, cheese |
| 16 | Strawberry, watermelon, rose, fish* | Strawberry, orange, rose, fish* |
| PILOT 4 - here only 2 items were changed as discriminated below | | |
| Item number | **Pilot 3** | **Pilot 4** |
| 2 | Leather*, milk, chocolate cake, popcorn | Leather*, milk, banana, popcorn |
| 12 | Butter, clove*, Brazilian baguette, guava | Butter, clove*, banana, guava |

* = Correct alternatives for items are marked by a star.
