# Supplementary material for: Effects of Age and Cognition on a Cross-Cultural Paediatric Adaptation of the Sniffin' Sticks Identification Test
Source: PLoS One. 2015 Aug 12;10(8):e0131641. doi: 10.1371/journal.pone.0131641 (PMC4534354; doi:10.1371/journal.pone.0131641)
Supplement: S2 Table — *Correct items. (DOCX) [file pone.0131641.s003.docx]

**S2 Table** - Portuguese version of SS16-Child

| Itens do SS16 | Opções | | | |
| --- | --- | --- | --- | --- |
| 1 | Queijo | Azeitona | Laranja* | Cebola |
| 2 | Couro* | Leite | Pipoca | Banana |
| 3 | Canela* | Batata Frita | Peixe | Leite com chocolate |
| 4 | Bolacha de água e sal | Azeitona | Menta* | Cebola |
| 5 | Hamburger | Banana* | Churrasco | Café |
| 6 | Pastel | Pão | Pipoca | Limão* |
| 7 | Bolo de chocolate | Sabonete de erva doce* | Batata Frita | Pipoca |
| 8 | Churrasco | Bolacha | Pão de Queijo | Tinta* |
| 9 | Alho* | Shampoo | Maçã | Mamão |
| 10 | Pastel | Mamão | Gasolina | Café* |
| 11 | Churrasco | Alho | Maçã* | Peixe |
| 12 | Manteiga | Cravo* | Banana | Guava |
| 13 | Manteiga | Abacaxi* | Queijo | Hamburger |
| 14 | Limão | Pastel | Batata Frita | Rosa* |
| 15 | Hamburger | Bolo de chocolate | Pasta de dente* | Queijo |
| 16 | Morango | Laranja | Rosa | Peixe* |

*Correct items
