# Supplementary material for: Effects of Age and Cognition on a Cross-Cultural Paediatric Adaptation of the Sniffin' Sticks Identification Test
Source: PLoS One. 2015 Aug 12;10(8):e0131641. doi: 10.1371/journal.pone.0131641 (PMC4534354; doi:10.1371/journal.pone.0131641)
Supplement: S3 Table — Legend: f—female; m = male; A—family income less than 5 Brazilian minimum wage; B—family income over than 5 Brazilian minimum wage. (DOCX) [file pone.0131641.s004.docx]

**S3 Table. Demographic data of 51 children participating in the final study**

| Identification | Sex | Age | Rhinitis | Smoke | Family income |
| --- | --- | --- | --- | --- | --- |
| 1 | F | 3 | no | no | A |
| 2 | M | 4 | no | no | A |
| 3 | F | 5 | no | no | A |
| 4 | F | 6 | no | no | A |
| 5 | F | 6 | no | no | A |
| 6 | F | 6 | no | no | A |
| 7 | M | 7 | no | no | B |
| 8 | F | 10 | no | no | A |
| 9 | M | 13 | no | no | A |
| 10 | F | 14 | yes | no | B |
| 11 | F | 14 | no | no | B |
| 12 | M | 12 | no | no | A |
| 13 | F | 9 | no | no | A |
| 14 | M | 9 | no | no | B |
| 15 | M | 5 | yes | no | A |
| 16 | F | 5 | yes | no | A |
| 17 | F | 14 | no | no | A |
| 18 | F | 15 | no | no | A |
| 19 | F | 12 | no | no | A |
| 20 | F | 6 | yes | no | A |
| 21 | M | 7 | no | no | A |
| 22 | F | 12 | no | no | A |
| 23 | F | 12 | no | no | A |
| 24 | F | 12 | yes | no | A |
| 25 | F | 10 | yes | no | A |
| 26 | F | 13 | yes | no | A |
| 27 | M | 15 | no | no | A |
| 28 | M | 11 | yes | no | A |
| 29 | F | 10 | no | no | A |
| 30 | F | 14 | no | no | A |
| 31 | F | 17 | yes | no | A |
| 32 | F | 17 | no | no | B |
| 33 | F | 15 | no | no | A |
| 34 | F | 8 | no | no | A |
| 35 | F | 5 | no | no | A |
| 36 | F | 11 | no | no | A |
| 37 | M | 8 | no | no | A |
| 38 | F | 10 | no | no | B |
| 39 | F | 9 | no | no | A |
| 40 | F | 8 | no | no | A |
| 41 | F | 6 | no | no | A |
| 42 | F | 6 | yes | no | A |
| 43 | F | 6 | no | no | A |
| 44 | M | 4 | no | no | A |
| 45 | M | 4 | no | no | B |
| 46 | M | 4 | no | no | A |
| 47 | F | 16 | no | no | B |
| 48 | M | 18 | yes | no | A |
| 49 | M | 15 | no | no | A |
| 50 | M | 16 | no | no | A |
| 51 | F | 15 | yes | no | A |

Legend: f - female; m = male; A - family income less than 5 Brazilian minimum wage; B - family income over than 5 Brazilian minimum wage
