# Supplementary material for: Effects of Age and Cognition on a Cross-Cultural Paediatric Adaptation of the Sniffin' Sticks Identification Test
Source: PLoS One. 2015 Aug 12;10(8):e0131641. doi: 10.1371/journal.pone.0131641 (PMC4534354; doi:10.1371/journal.pone.0131641)
Supplement: S6 Table — The columns show the percentage of subjects in each age group who correctly identified each item. Cells are coloured according to the following rules: ≥75% green, 60–74.9% yellow, <60% red. N = number of subjects; y = years. (DOCX) [file pone.0131641.s007.docx]

# S6 Table - Percentage of correct identification by age group

| **Age**  **Group** | **Orange** | **Leather** | **Cinnamon** | **Mint** | **Banana** | **Lemon** | **Fennel**  **Soap** | **Paint** | **Garlic** | **Coffee** | **Apple** | **Clove** | **Pineapple** | **Rose** | **Toothpaste** | **Fish** |
| --- | --- | --- | --- | --- | --- | --- | --- | --- | --- | --- | --- | --- | --- | --- | --- | --- |
| **3-5.99y (N=9)** | **77,8%** | **44,4%** | **11,1%** | **55,6%** | **88,9%** | **77,8%** | **44,4%** | **33,3%** | **66,7%** | **66,7%** | **77,8%** | **55,6%** | **77,8%** | **55,6%** | **66,7%** | **66,7%** |
| **6-9.99y (N=15)** | **93,3%** | **66,7%** | **60,0%** | **80,0%** | **93,3%** | **100%** | **66,7%** | **73,3%** | **100%** | **93,3%** | **80,0%** | **93,3%** | **100%** | **100%** | **86,7%** | **100%** |
| **10-13.99y (N=13)** | **100%** | **100%** | **91,7%** | **100%** | **91,7%** | **100%** | **91,7%** | **100%** | **100%** | **91,7%** | **83,3%** | **83,3%** | **91,7%** | **91,7%** | **100%** | **91,7%** |
| **14-18y (N=14)** | **100%** | **92,9%** | **100%** | **92,9%** | **100%** | **92,9%** | **100%** | **100%** | **100%** | **100%** | **100%** | **100%** | **100%** | **100%** | **92,9%** | **92,9%** |

The columns show the percentage of subjects in each age group who correctly identified each item. Cells are coloured according to the following rules: ≥75% green, 60-74.9% yellow, <60% red.

N= number of subjects; y=years
