# Supplementary material for: Effects of Age and Cognition on a Cross-Cultural Paediatric Adaptation of the Sniffin' Sticks Identification Test
Source: PLoS One. 2015 Aug 12;10(8):e0131641. doi: 10.1371/journal.pone.0131641 (PMC4534354; doi:10.1371/journal.pone.0131641)
Supplement: S7 Table — Legend: f—female; m = male; A—family income less than 5 Brazilian minimum wage; B—family income over than 5 Brazilian minimum wage. (DOCX) [file pone.0131641.s008.docx]

The tables S7, S8 and S9 contain the raw data for all adult subjects tested. As seen from this data, the performance of adults in this test which was designed for children aged as young as 3 years old is not surprisingly very good.

**S7 Table. Demographic data of adults**

| **Identification** | **Sex** | **Age** | **Rhinitis** | **Smoke** | **Family income** |
| --- | --- | --- | --- | --- | --- |
| 1 | f | 43 | no | yes | A |
| 2 | f | 32 | no | no | A |
| 3 | m | 34 | no | no | A |
| 4 | m | 51 | no | yes | A |
| 5 | m | 22 | yes | no | A |
| 6 | f | 46 | no | no | A |
| 7 | m | 58 | no | yes | B |
| 8 | f | 36 | no | no | A |
| 9 | m | 26 | no | no | A |
| 10 | f | 48 | no | no | A |
| 11 | f | 33 | no | no | A |
| 12 | f | 37 | no | yes | A |
| 13 | m | 49 | no | yes | A |
| 14 | f | 58 | yes | yes | A |
| 15 | m | 24 | yes | no | A |
| 16 | m | 29 | no | no | A |
| 17 | m | 35 | no | no | B |
| 18 | f | 49 | no | yes | A |
| 19 | f | 44 | yes | no | A |
| 20 | f | 34 | yes | no | A |

Legend: f - female; m = male; A - family income less than 5 Brazilian minimum wage; B - family income over than 5 Brazilian minimum wage
